# Aberrant basement membrane production by HSCs in MASLD is attenuated by the bile acid analog INT-767

**DOI:** 10.1097/HC9.0000000000000574

**Published:** 2024-11-25

**Authors:** Prakash Ramachandran, Madara Brice, Elena F. Sutherland, Anna M. Hoy, Eleni Papachristoforou, Li Jia, Frances Turner, Timothy J. Kendall, John A. Marwick, Neil O. Carragher, Denise Oro, Michael Feigh, Diana J. Leeming, Mette J. Nielsen, Morten A. Karsdal, Nadine Hartmann, Mary Erickson, Luciano Adorini, Jonathan D. Roth, Jonathan A. Fallowfield

**Affiliations:** 1 Centre for Inflammation Research, Institute for Regeneration and Repair, University of Edinburgh, Edinburgh, UK; 2 Edinburgh Genomics, University of Edinburgh, Edinburgh, UK; 3 Edinburgh Pathology, University of Edinburgh, Edinburgh, UK; 4 Cancer Research UK Scotland Centre, Institute of Genetics and Cancer, University of Edinburgh, Edinburgh, UK; 5 Gubra, Hørsholm, Denmark; 6 Nordic Bioscience, Herlev, Denmark; 7 Intercept Pharmaceuticals Inc., San Diego, California, USA

**Keywords:** basement membrane, extracellular matrix, Farnesoid X receptor, INT-767, liver fibrosis, metabolic dysfunction–associated steatotic liver disease, NASH

## Abstract

**Background:**

The farnesoid X receptor (FXR) is a leading therapeutic target for metabolic dysfunction–associated steatohepatitis (MASH)-related fibrosis. INT-767, a potent FXR agonist, has shown promise in preclinical models. We aimed to define the mechanisms of INT-767 activity in experimental MASH and dissect cellular and molecular targets of FXR agonism in human disease.

**Methods:**

Leptin-deficient *ob*/*ob* mice were fed a MASH-inducing diet for 15 weeks before the study started. After baseline liver biopsy and stratification, mice were allocated to INT-767 (10 mg/kg/d) or vehicle treatment for 8 weeks, either alongside an ongoing MASH diet (progression) or following conversion to normal chow (reversal). Effects on extracellular matrix remodeling were analyzed histologically and by RNA-sequencing. Serum fibrosis biomarkers were measured longitudinally. Human liver samples were investigated using bulk and single-cell RNA-sequencing, histology, and cell culture assays.

**Results:**

INT-767 treatment was antifibrotic during MASH progression but not reversal, attenuating the accumulation of type I collagen and basement membrane proteins (type IV collagen and laminin). Circulating levels of PRO-C4, a type IV collagen formation marker, were reduced by INT-767 treatment and correlated with fibrosis. Expression of basement membrane constituents also correlated with fibrosis severity and adverse clinical outcomes in human MASH. Single-cell RNA-sequencing analysis of mouse and human livers, and immunofluorescence staining colocalized FXR and basement membrane expression to myofibroblasts within the fibrotic niche. Treatment of culture-activated primary human HSCs with INT-767 decreased expression of basement membrane components.

**Conclusions:**

These findings highlight the importance of basement membrane remodeling in MASH pathobiology and as a source of circulating biomarkers. Basement membrane deposition by activated HSCs is abrogated by INT-767 treatment and measurement of basement membrane molecules should be included when determining the therapeutic efficacy of FXR agonists.

## INTRODUCTION

The growing global health and economic burden of metabolic dysfunction–associated steatohepatitis (MASH) is well-documented,^1^ but there is only 1 approved pharmacological therapy for this disease (resmetirom) which improved key readouts of liver histopathology in just 25%–30% of patients.^2^ Targeting fibrosis in patients with MASH is crucial as fibrosis stage is associated with all-cause mortality and liver-related morbidity and mortality.^3^ HSCs play a pivotal role in the initiation, progression, and regression of hepatic fibrosis.^4^ Following chronic liver injury, quiescent HSCs transdifferentiate into proliferative, migratory, contractile myofibroblasts that secrete abundant extracellular matrix (ECM) structural and remodeling proteins and serve as a hub of intrahepatic signaling through HSC-derived stellakines.^5^ Consequently, HSC-derived myofibroblasts and the ECM itself are leading targets for antifibrotic pharmacotherapies. It is well established that activated HSCs are a key source of fibrillar collagen (types I, III, and V) production in advanced hepatic fibrosis/cirrhosis and that these ECM components increase up to 10-fold relative to liver dry weight.^6^ Other ECM components are also altered significantly in fibrotic liver tissue, with aberrant basement membrane deposition increasingly recognized as an important pathological feature in chronic liver disease.^7^^,^^8^ Indeed, basement membrane collagen (type IV) increases by up to 6-fold in liver fibrosis,^6^^,^^9^ while the accumulation of type IV collagen and other basement membrane ECM components within the space of Disse leads to capillarization of the sinusoids and impairing organ function.^10^ Elevated serum type IV collagen and laminin levels are related to the degree of fibrosis in MASH and other chronic liver diseases^10^^–^^12^ and basement membrane proteins may also predict disease progression and survival in patients with early-stage cirrhosis.^7^ Furthermore, knockout of minor type IV collagen a5 chain ameliorates experimental liver fibrosis by reducing HSC activation and promoting hepatocyte proliferation.^7^ Despite emerging evidence of an important role in liver fibrosis progression, basement membrane components are not commonly assessed when studying the efficacy of antifibrotic interventions in MASH.

The farnesoid X receptor (FXR; NR1H4), a member of the nuclear hormone superfamily, is a ligand-activated transcription factor expressed in the liver, intestine, kidney, adipose, and other tissues that is a key regulator of bile acid, inflammatory, fibrotic, and metabolic pathways. FXR agonism is an established approach for improving MASH histological endpoints, including fibrosis, and several steroidal and nonsteroidal FXR agonists have advanced to phase II/III clinical trials in MASH as monotherapies or as the backbone of combination drug regimens.^13^^,^^14^ The first-in-class selective FXR agonist obeticholic acid (OCA; INT-747 [6a-ethyl-chenodeoxycholic acid]) improved fibrosis by ≥1 stage (with no worsening of NASH) in 22.4% of patients in the high-dose arm (vs. 9.6% receiving placebo; *p* < 0.0001) in the REGENERATE phase III clinical trial (ClinicalTrials.gov Identifier: NCT02548351).^13^ However, it is still unclear how FXR agonists mediate an antifibrotic effect in MASH, which specific ECM molecules are modulated, and whether HSCs are the main cellular target. Indeed, while some previous studies have demonstrated FXR expression and a functional effect of FXR modulation on HSC phenotype,^15^^,^^16^ other groups have failed to detect functional levels of FXR and FXR pathway activity in isolated HSCs,^17^ with no demonstrable effect of OCA on rodent or human HSC activation.^18^ Hence, it remains unclear what specific impacts FXR agonists have on HSC phenotype and ECM production.

The semi-synthetic bile acid analog INT-767 (6a-ethyl-24-nor-5b-cholane-3 a-,7 a-,23-triol-23 sulfate sodium salt) is a dual agonist of FXR and the Takeda-G-protein-receptor-5 (TGR5; GPBAR1).^19^ It is a 3-fold more potent FXR agonist than OCA (EC_50_ 30 nM vs. 99 nM) and has shown antifibrotic effects in hepatic cell culture models^20^ and in diet-induced mouse models of MASH.^21^^,^^22^ Indeed, INT-767 has greater antifibrotic and antisteatotic effects than OCA in genetically obese (leptin-deficient *ob/ob*) MASH mice.^21^ INT-767 treatment also inhibits liver inflammation and biliary fibrosis in MDR2-deficient mice, while the FXR-specific OCA and TGR5-specific compound INT-777 (6a-ethyl-23(S)-methyl-cholic acid) has no protective effects.^23^ Furthermore, INT-767 reduces liver triglyceride and cholesterol ester accumulation and modulates bile acid composition in murine MASH,^22^ while also exerting broader metabolic effects such as promoting visceral fat brown adipogenesis and mitochondrial function.^24^ In a high-fat feeding mouse model, INT-767 reversed obesity, dependent on activation of both TGR5 and FXR and reversed the development of atherosclerosis and MASH.^25^ Thus, treatment with INT-767 could represent an attractive strategy for the treatment of chronic liver disease in people with MASH and metabolic comorbidities. However, the antifibrotic mechanisms and cellular targets of INT-767 in the injured liver remain unclear.

In this study, we aimed to investigate the underlying mechanisms of INT-767 antifibrotic activity, using a validated mouse model of Amylin Liver NASH (AMLN) diet-induced and biopsy-confirmed MASH.^26^^,^^27^ We demonstrate the striking effects of INT-767 on basement membrane ECM production following chronic liver injury, most likely mediated through direct effects on activated HSCs. Subsequent target validation approaches in human liver tissues reinforce the potential of INT-767 as an antifibrogenic agent for the treatment of MASH-related fibrosis.

## METHODS

### *Ob/ob* mouse model of AMLN diet-induced and biopsy-confirmed fibrotic MASH

**FIGURE 1:**
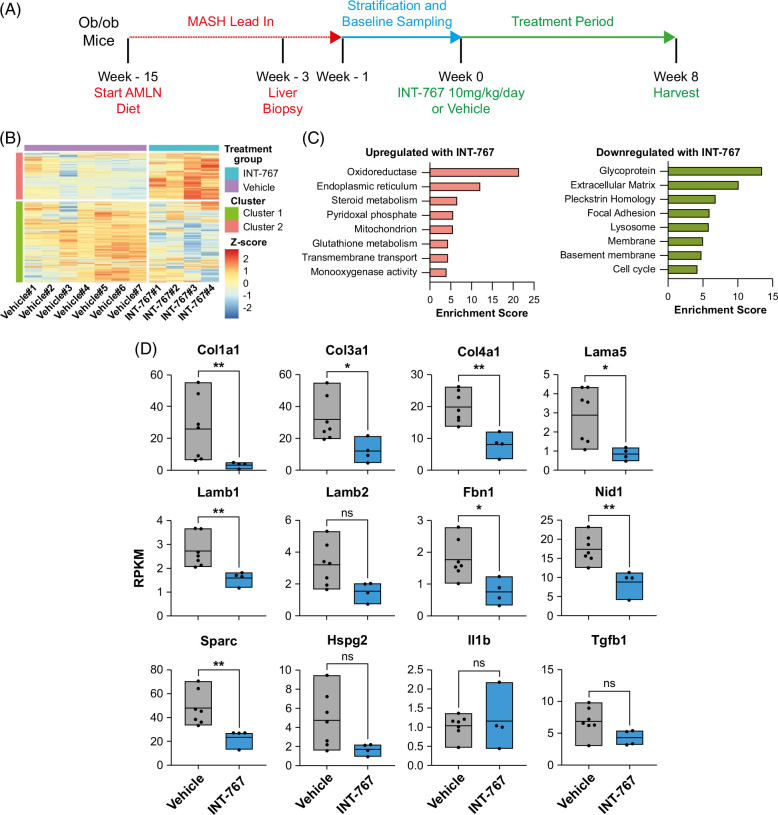
INT-767 treatment inhibits basement membrane expression in AMLN *ob/ob*-MASH mice. (A) Summary of AMLN *ob/ob*-MASH mouse model. Lep^ob/ob^ mice on an AMLN diet for 12 weeks underwent liver biopsy. Mice with fibrosis stage ≥1 and steatosis score ≥2 continued the AMLN diet and were randomized to treatment with either INT-767 (10 mg/kg) or vehicle once daily for a further 8 weeks. (B) RNA-seq analysis of liver tissue demonstrates modules of differentially expressed genes that are downregulated (cluster 1) and upregulated (cluster 2) following INT-767 treatment. (C) Annotation of statistically significantly enriched functional categories of genes in cluster 1 and cluster 2 using the DAVID tool identifies the downregulation of extracellular matrix and basement membrane genes following INT-767 treatment. (D) Comparison of expression levels of stated gene between the vehicle (n = 7) and INT-767 (n = 4) treated mice using RNA-seq data. Levels of significance: *p < 0.05, **p < 0.01, ns *p* ≥ 0.05 (Mann-Whitney test). Abbreviations: AMLN, Amylin Liver NASH; MASH, metabolic dysfunction–associated steatohepatitis; RPKM, reads per kilobase of transcript per million mapped reads.

Male, 5–6-week-old Lep^*ob/ob*^ mice (B6.V-Lep^*ob*^/JRj [*ob/ob*]; Janvier Labs) were fed the AMLN diet high in fat (40% containing 18% trans-fat), 40% carbohydrate (20% fructose), and 2% cholesterol (#D09100301; Research Diets Inc) for 15 weeks before and throughout the drug treatment schedule (Figure 1A). All AMLN *ob/ob*-MASH mice underwent baseline wedge liver biopsy before treatment, as described.^21^ A priori histopathological inclusion criteria in subsequent treatment arms were a steatosis score ≥2 and a fibrosis stage score ≥1 as evaluated by 1 pathologist using the clinical criteria outlined by Kleiner et al.^28^ Animals were single-housed after the biopsy procedure. Animals were stratified (*n* = 10–12 per group) based on mean fibrosis area, as assessed by hepatic type I collagen immunostaining, to equally distribute animals with similar fibrosis between treatment groups.^26^

### Drug treatment of AMLN *ob/ob*-MASH mouse model

INT-767 (Intercept Pharmaceuticals Inc) was dissolved in 0.5% carboxymethyl cellulose (CMC) for the dosing volume of 5 mL/kg. After AMLN diet induction and stratification, *ob/ob*-MASH mice were randomized to oral gavage administration of INT-767 (10 mg/kg) or vehicle (0.5% CMC) once daily for 8 weeks. Body weight was monitored once daily during the intervention.

### Combined chow-reversal and drug treatment of AMLN *ob/ob*-MASH mouse model

INT-767 was dissolved in 0.5% CMC for dosing volume of 5 mL/kg. After AMLN diet induction (15 wk) and stratification based on biopsy, *ob/ob*-MASH mice were randomized to oral gavage administration of INT-767 (10 mg/kg) or vehicle (0.5% CMC) once daily for 8 weeks with animals concomitantly shifted to normal chow (Altromin 1324, Altromin Spezialfutter GmbH & Co KG) (Supplemental Figure S3, http://links.lww.com/HC9/B86). Body weight was monitored once daily during the intervention.

### Liver histology

Baseline liver biopsy and terminal samples (both left lateral lobe) were fixed in 4% paraformaldehyde, paraffin-embedded and sectioned (3 μm thickness) before staining with hematoxylin and eosin and antibodies to type I collagen, type IV collagen, and laminin using standard techniques. Primary antibodies are described in Supplemental Table S1, http://links.lww.com/HC9/B86. The nonalcoholic fatty liver disease activity score (NAS) and NASH Clinical Research Network fibrosis staging system were applied for the assessment of steatosis (score 0–3), lobular inflammation (score 0–3), hepatocyte ballooning (score 0–2), and fibrosis (stage 0–4). In liver sections stained for type I collagen, type IV collagen, and laminin, histomorphometry was applied using digital imaging software (Visiomorph, Visiopharm). The fractional area of liver fat (macrosteatosis) was determined on hematoxylin and eosin–stained sections and expressed relative to the total sectional area. The fractional area of type I collagen, type IV collagen, and laminin immunostaining were expressed relative to the total parenchymal area by subtracting the corresponding fat area determined on adjacent hematoxylin and eosin–stained sections. All histological assessments were performed by histopathologists blinded to the experimental groups.

Anonymized human liver samples were supplied after approval by the National Health Service Research Scotland (NRS) biorepository network (East of Scotland Research Ethics Service REC 1 [Reference: 15/ES/0094]). Formalin-fixed paraffin-embedded human metabolic dysfunction–associated steatotic liver disease (MASLD) cirrhotic liver tissue was sectioned (4 µm thickness). Slides were dewaxed and rehydrated by incubation in xylene for 2 × 5 minutes followed by 100%, 75%, and 65% ethanol for 2 minutes each. Slides were incubated with pepsin (#R2283; Sigma-Aldrich) for 10 minutes at 37°C, then transferred to a Bond RX Automated Research Stainer (Leica). After 10 minutes of hydrogen peroxide (diluted 1:10 in water) blocking, 3 sequential rounds of staining were performed. Each round consisted of 30 minutes blocking (#ab64226; Abcam), 60 minutes primary antibody incubation, 30 minutes ImPRESS HRP polymer incubation according to species of primary antibody (rabbit: #MP-7401-50 and goat: #MP-7405-50; Vector Laboratories, Upper Heyford), and 10 minutes fluorescent tyramide signal amplification reagent incubation (Cy3: #NEL744001KT, Cy5: #NEL745001KT, and FITC: #NEL741001KT; Akoya Biosciences). Primary antibodies are described in Supplemental Table S1, http://links.lww.com/HC9/B86. The antibody complex was stripped in between rounds by incubation in Leica Bond ER1 buffer for 20 minutes at 99°C. Following the 3 sequential rounds, slides were incubated in DAPI for 10 minutes and mounted using ProLong Gold antifade reagent (ThermoFisher Scientific). Slides were imaged using an AxioScan.Z1 slide scanner at ×20 magnification.

### Statistical analysis

Unless stated, all data are presented as mean ± SE. Differences between multiple groups were assessed using Welch’s ANOVA with the Dunnett T3 multiple comparison test, 2-way ANOVA, or repeated measures ANOVA, and between pairs with an unpaired 2-tailed Student *t* test (if normally distributed) or a Mann-Whitney test (if not normally distributed). Correlations were performed using simple linear regression. GraphPad Prism version 9.2.0 (GraphPad Software Inc) was used to perform statistical calculations, and *p* < 0.05 was considered statistically significant.

### Data accessibility

Human bulk RNA-seq data are deposited in the European Nucleotide Archive (PRJEB58625),^29^ and mouse RNA-seq data in the Gene Expression Omnibus (GSE275813).

### Supplemental methods

Methods for serum analysis, transcriptomics analysis, and cell culture are provided in Supplemental Digital Content, http://links.lww.com/HC9/B86. qPCR primers are listed in Supplemental Table S2, http://links.lww.com/HC9/B86.

## RESULTS

### INT-767 treatment modulates ECM expression pathways in AMLN *ob/ob*-MASH mice

To explore the mechanisms by which INT-767 mediates beneficial effects in MASH, we performed unbiased RNA-seq on whole-liver samples taken from AMLN diet-induced and biopsy-confirmed *ob/ob*-MASH mice treated with either INT-767 (n = 4) or vehicle (n = 7) once daily for 8 weeks (Figure 1A). We identified modules of genes which were significantly upregulated (cluster 2) and downregulated (cluster 1) in the liver following INT-767 administration to mice with MASH (Figure 1B, Supplemental Table S3, http://links.lww.com/HC9/B87). As expected, the expression of FXR target genes *Nr0b2* (SHP), *Abcb11* (BSEP), and *Apoa5* ^21^^,^^30^^,^^31^ was significantly increased in INT-767–treated mice. Consistent with previous literature,^22^^,^^32^ INT-767 resulted in altered liver expression of key genes involved in bile synthesis (*Cyp7a1* and *Cyp8b1*), excretion (*Abcb11*), resorption (*Slco1a4*/Oatp2), and efflux (*Slc51b*/Ostb) (Supplemental Figure S1A, http://links.lww.com/HC9/B86). In keeping with the role of FXR in lipid homeostasis, INT-767 increased hepatic gene expression of key molecules *Scarb1*, *Lipc*, and *Lcat* with a trend to increased *Hmgca* and reduced expression of *Scd1* and *Vldlr* ^31^^,^^33^ (Supplemental Figure S1A, http://links.lww.com/HC9/B86). No difference was observed in hepatic *Fgf15* or *Fgfr4* expression (data not shown). To assess the potential antifibrotic mechanisms of INT-767, we performed functional annotation clustering analysis on the modules of significantly differentially expressed genes (Figure 1C). INT-767 treatment in murine MASH resulted in the upregulation of genes related to liver metabolic functions, while there was a significant reduction in the expression of ECM components in keeping with antifibrotic effects (Figure 1C). Notably among the most downregulated functional gene clusters were glycoproteins and basement membrane components, which are increasingly recognized as key pathological features of chronic liver disease.^10^

Given the striking overall reduction in ECM and basement membrane components following treatment with INT-767 (Figure 1C), we examined alterations in individual ECM molecules using our RNA-seq data. We noted a significant reduction in gene expression of fibrillar collagens *Col1a1* and *Col3a1* (Figure 1D), indicating an antifibrogenic mechanism for INT-767.^21^ Furthermore, we noted significant downregulation of several key glycoproteins and basement membrane components including *Col4a1*, *Lama5*, *Lamb1*, *Fbn1, Nid1*, and *Sparc* (Figure 1D). No differences were observed in the expression of proinflammatory and profibrotic cytokines, suggesting that INT-767 may have a direct role in regulating ECM and basement membrane molecule production in experimental MASH.

### INT-767 treatment reduces basement membrane production in AMLN *ob/ob*-MASH mice

Having defined significant alterations in hepatic gene expression profiles following INT-767 treatment, we performed a more detailed phenotypic assessment of the effects of INT-767 in AMLN *ob/ob*-MASH mice. Drug treatment did not affect food intake (Supplemental Figure S1B, http://links.lww.com/HC9/B86) or body weight (Supplemental Figure S1C, http://links.lww.com/HC9/B86), but as anticipated^21^ we detected a reduction in serum total cholesterol, with no difference in triglycerides (Supplemental Figure S1D, http://links.lww.com/HC9/B86).

**FIGURE 2:**
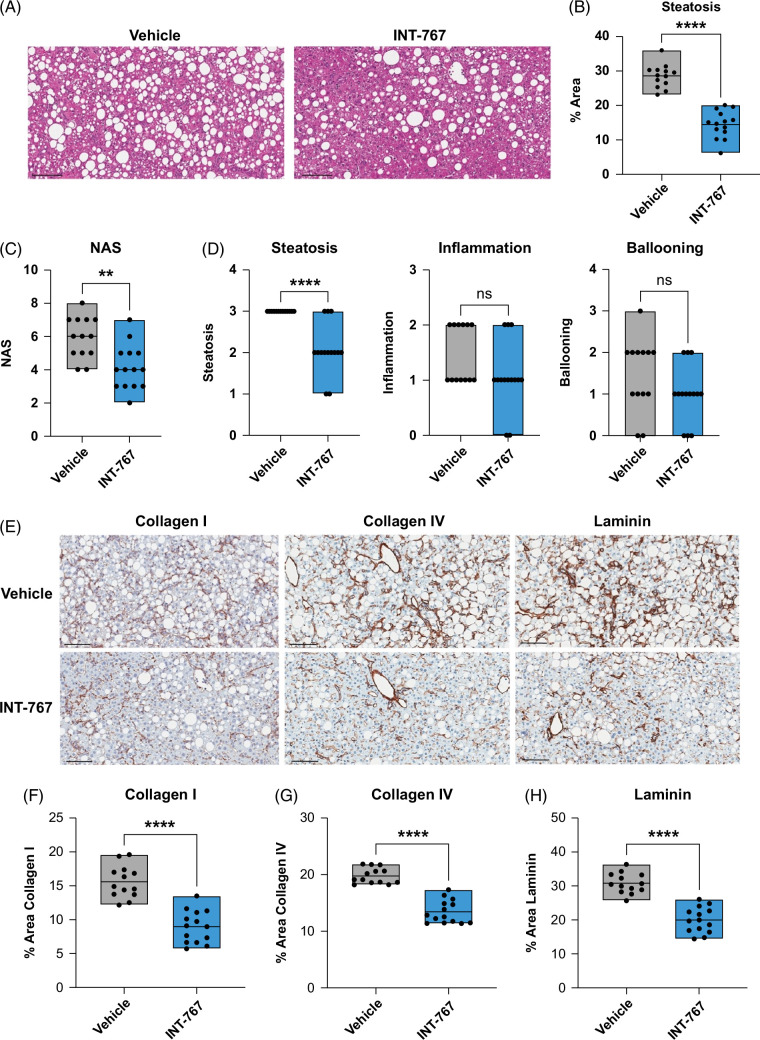
INT-767 treatment reduces basement membrane deposition in AMLN *ob/ob*-MASH mice. (A) Representative liver H&E images of AMLN *ob/ob*-MASH mouse model following treatment with INT-767 or vehicle. Scale bar = 100 μm. (B) Histological quantification of % steatosis in AMLN *ob/ob*-MASH mice following treatment with INT-767 (n = 14) or vehicle (n = 13). Levels of significance: ****p < 0.0001 (unpaired *t* test). (C) Histological quantification of NAS in AMLN *ob/ob*-MASH mice following treatment with INT-767 (n = 14) or vehicle (n = 13). Levels of significance: **p = 0.0024 (Mann-Whitney test). (D) Histological quantification of NAS components in AMLN *ob/ob*-MASH mice following treatment with INT-767 (n = 14) or vehicle (n = 13). Levels of significance: ****p < 0.0001, ns (inflammation) *p* = 0.14, ns (ballooning) *p* = 0.13 (Mann-Whitney test). (E) Representative liver immunohistochemistry images of AMLN *ob/ob*-MASH mice following treatment with INT-767 or vehicle for stated protein. Scale bar = 100 μm. (F–H) Histological quantification of % type I collagen, type IV collagen, and laminin staining in AMLN *ob/ob*-MASH mice following treatment with INT-767 (n = 14) or vehicle (n = 13). Levels of significance: ****p < 0.0001 (unpaired *t* test). Abbreviations: AMLN, Amylin Liver NASH; H&E, hematoxylin and eosin; MASH, metabolic dysfunction–associated steatohepatitis; NAS, nonalcoholic fatty liver disease activity score.

Histological assessment of the liver demonstrated significantly reduced liver steatosis following INT-767 treatment (Figures 2A, B), also reflected by significant reductions in liver:body weight ratio (Supplemental Figure S1E, http://links.lww.com/HC9/B86) and the NAS (Figures 2C, D). However, no significant effects were observed on serum AST or ALT levels (Supplemental Figure S1E, http://links.lww.com/HC9/B86) or on histological liver inflammation scores (Figure 2D).

Previous work in AMLN *ob/ob*-MASH mice showed a reduction in total liver scar (assessed by tinctorial staining using picrosirius red) following treatment with INT-767.^21^ To assess changes in specific ECM components in MASH, we performed histological quantification of type I collagen, type IV collagen, and laminin, having excluded fat to account for effects of INT-767 on liver steatosis (Figure 2E). In keeping with our RNA-seq analysis, INT-767 treatment resulted in a marked reduction in liver type I collagen staining (Figure 2F). Basement membrane integrity was also improved, evidenced by reduced type IV collagen (13.44 ± 0.55% [INT-767] vs. 19.82 ± 0.39% [vehicle]; *p* < 0.0001) (Figure 2G) and laminin (19.98 ± 1.0% [INT-767] vs. 30.78 ± 0.83% [vehicle]; *p* < 0.0001) (Figure 2H) deposition compared with controls. To assess the magnitude of treatment effects, we compared the posttreatment liver histology (8 wk) with baseline (−3 wk) liver biopsies for each animal. Of note, the degree of steatosis declined with MASH progression, and this reduction was accentuated with INT-767 treatment (Supplemental Figure S2A, http://links.lww.com/HC9/B86). Hepatic type I collagen and type IV collagen deposition progressively increased with ongoing liver injury and vehicle treatment, but this increase was abrogated by INT-767 treatment (Supplemental Figure S2A, http://links.lww.com/HC9/B86). Hence, INT-767 therapy can prevent liver ECM accumulation during MASH progression.

Accumulation of ECM in the chronically injured liver reflects the balance of production and degradation of diverse molecules within the parenchyma.^34^ The effects of INT-767 on liver gene expression suggest reduced basement membrane production (Figure 1D). To confirm this, we performed serial measurements of circulating PRO-C4,^35^ a serum biomarker of type IV collagen production which is increased in patients with MASH.^36^ Serum PRO-C4 (ng/mL) increased during MASH-induction from 85.9 ± 3.6 at baseline to 152.4 ± 3.6 at 8 weeks in vehicle-treated mice (Supplemental Figure S2B, http://links.lww.com/HC9/B86). INT-767 treatment significantly attenuated the increase in circulating levels of PRO-C4 during MASH-induction (Supplemental Figure S2B, http://links.lww.com/HC9/B86), with a trend to reduced circulating levels of C4M, a type IV collagen degradation product^37^ (Supplemental Figure S2C, http://links.lww.com/HC9/B86). Overall, these findings confirm that INT-767 therapy inhibits type IV collagen production in experimental MASH. In addition, the observed reduction in PRO-C4 not only paralleled INT-767 antifibrotic efficacy but also correlated strongly with type I collagen, type IV collagen, and laminin tissue content (Supplemental Figure S2D, http://links.lww.com/HC9/B86), emphasizing its utility as a circulating biomarker of hepatic ECM deposition in MASH.

### Combined INT-767 treatment and chow-reversal does not accelerate ECM degradation in AMLN *ob/ob*-MASH mice

We next assessed whether INT-767 would be additive to histological improvements under conditions of fibrosis regression elicited by dietary lifestyle intervention (commonly recommended for patients with MASH). AMLN diet-induced and biopsy-confirmed *ob/ob*-MASH mice were transitioned from the AMLN induction diet to a lean control diet and treated with either vehicle or INT-767 for 8 weeks (Supplemental Figure S3A, http://links.lww.com/HC9/B86). In this context, while there was no significant overall effect of INT-767 treatment on NAS improvement (Supplemental Figure S3B, http://links.lww.com/HC9/B86), drug therapy accelerated steatosis regression (Supplemental Figure S3C, http://links.lww.com/HC9/B86). Focussing on the ECM components, INT-767 did not enhance type I collagen (Supplemental Figure S3D, http://links.lww.com/HC9/B86) or type IV collagen (Supplemental Figure S3E, http://links.lww.com/HC9/B86) regression, although a significant reduction in hepatic laminin was observed (Supplemental Figure S3F, http://links.lww.com/HC9/B86). Furthermore, during MASH regression, no significant differences in circulating PRO-C4 levels were seen in response to INT-767 treatment (Supplemental Figure S3G, http://links.lww.com/HC9/B86) with a minor reduction in C4M observed (Supplemental Figure S3H, http://links.lww.com/HC9/B86). Taken together, these data highlight that the predominant antifibrotic effects of INT-767 in experimental MASH are due to reduced ECM production during ongoing injury.

### Defining basement membrane responses in human MASH

**FIGURE 3:**
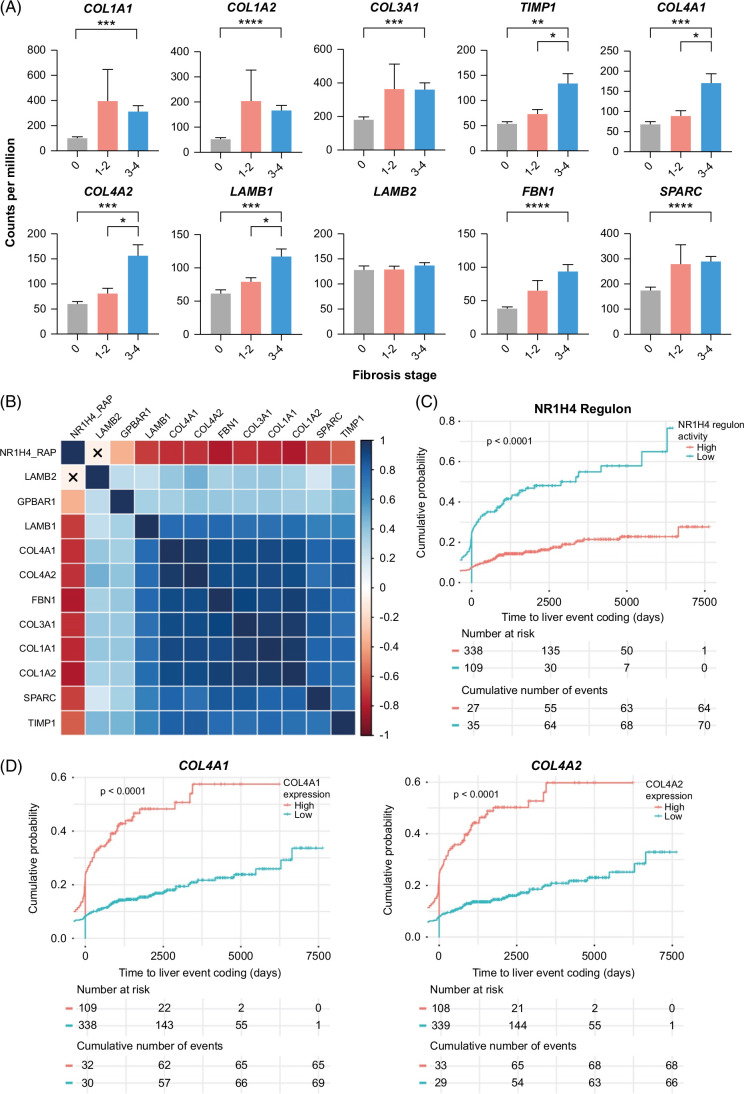
Basement membrane deposition in the fibrotic niche of human MASH. (A) RNA-seq analysis of human MASH liver tissue, grouped by histological NASH Clinical Research Network fibrosis stage. Comparison of expression (in counts per million) of stated gene between fibrosis stage 0 (n = 10), stage 1–2 (n = 19), and stage 3–4 (n = 25). Levels of significance: ****p < 0.0001, ***p < 0.001, **p < 0.01, *p < 0.05 (Welch’s ANOVA with Dunnett’s T3 multiple comparison test). (B) Correlation matrix of NR1H4 (FXR) regulon activity profile (NR1H4_RAP) and normalized gene expression counts for stated genes in the complete n = 663 set of cases with RNA-seq from the SteatoSITE data set. Statistically significant correlations (*p* < 0.05) shown as shaded boxes and nonsignificant correlations marked with an X. Boxes colored by Spearman correlation coefficients. (C, D) Biopsy cases from the SteatoSITE data set (n = 447) with available RNA-seq and no hepatic decompensation–related coding event before the time of the biopsy were used for time-to-event analysis. NR1H4 (FXR) regulon activity profile (C) and normalized gene expression counts for COL4A1 and COL4A2 (D) were used to divide cases into low and high risk of hepatic decompensation (Kaplan-Meier estimator curves with log-rank test *p* value shown). Abbreviation: MASH, metabolic dysfunction–associated steatohepatitis.

INT-767 treatment had a striking effect on basement membrane components in experimental MASH. While basement membrane components *COL4A2* and *LAMB1* were identified as prioritized genes specifically associated with liver fibrosis progression independent of etiology,^38^ no comprehensive assessment of basement membrane expression across the severity spectrum of human MASH has previously been performed. We performed bulk RNA-seq on 54 patient liver tissue specimens across the full severity spectrum of MASLD and compared gene expression profiles with detailed histological assessment. Basement membrane components, including *COL4A1*, *LAMB1*, and *FBN1*, were significantly upregulated as the histological fibrosis stage increased, mirroring fibrillar collagens and profibrogenic mediators such as *TIMP1* (Figure 3A). Furthermore, hepatic basement membrane gene expression in human MASLD showed a significant positive correlation with the degree of liver fibrosis as assessed by quantitative analysis of picrosirius red staining (collagen proportionate area) (Supplemental Figures S3A, B, http://links.lww.com/HC9/B86). These findings are similar to recent histological data in alcohol-associated liver disease, where type IV collagen is observed as an important component of perisinusoidal and pericellular fibrosis.^9^ In contrast, no increase in basement membrane gene expression was observed with worsening NAS (Supplemental Figure S4C, http://links.lww.com/HC9/B86) or liver steatosis (Supplemental Figures S4D, E, http://links.lww.com/HC9/B86).

To study the links between FXR activity and hepatic basement membrane expression in human MASLD, we performed additional analyses on RNA-seq data from liver biopsy samples across the full MASLD spectrum in the SteatoSITE cohort.^29^ Notably, significant inverse correlations were detected between hepatic FXR (NR1H4) regulon activity and expression of fibrillar collagens and basement membrane components in human MASLD (Figure 3B). In contrast, *TGR5* (GPBAR1) expression showed a weaker positive correlation with the expression of hepatic ECM components (Figure 3B). The SteatoSITE cohort also includes linked clinical outcome data.^29^ Strikingly, low hepatic FXR regulon activity and high expression of *COL4A1* and *COL4A2* were associated with increased rates of hepatic decompensation (Figures 3C, D) and higher overall mortality (Supplemental Figures S4F, G, http://links.lww.com/HC9/B86) in patients with MASLD. In summary, these data indicate that aberrant liver basement membrane responses are associated with worsening fibrosis, low hepatic FXR activity, and adverse clinical outcomes in patients with MASLD.

We have previously shown that pathogenic cell populations in fibrotic human liver tissue reside in a distinct spatial microenvironment termed the fibrotic niche.^39^ To spatially localize basement membrane components in fibrotic human MASH tissue, we performed multiplex immunofluorescence. Both type IV collagen and laminin were deposited in the fibrotic niche, in close proximity to fibrillar type I collagen (Supplemental Figure S5A, http://links.lww.com/HC9/B86). Type IV collagen and laminin deposition were also identified in liver lobules in a perisinusoidal pattern, although less collagen I was observed in this niche (Supplemental Figure S5B, http://links.lww.com/HC9/B86). Hence, basement membrane proteins contribute directly to the ECM architecture of the fibrotic niche in patients with MASLD.

### Activated HSCs are the likely in vivo target population for INT-767

**FIGURE 4:**
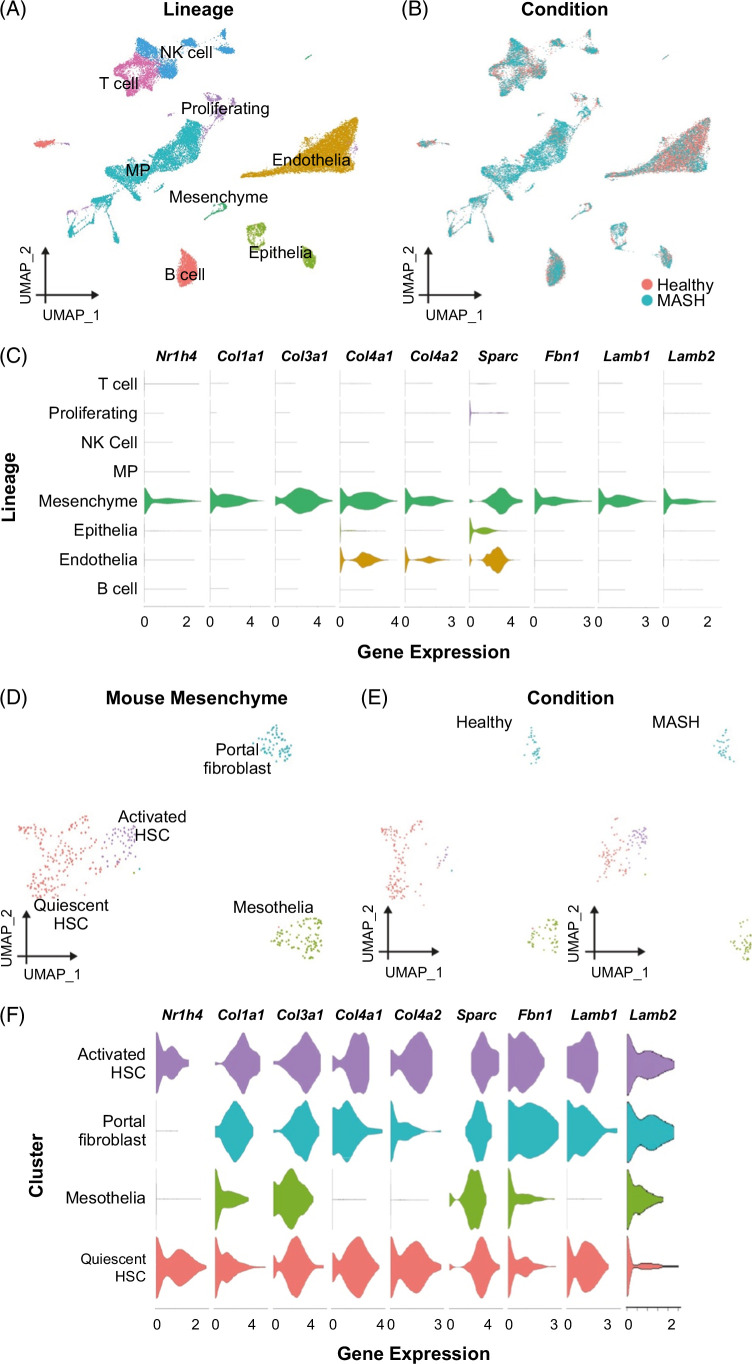
Activated HSCs are a candidate in vivo target population for INT-767 in murine MASH. (A) UMAP showing annotation of mouse liver scRNA-seq data (33,168 cells) by cell lineage. (B) UMAP showing whether cells are from healthy (n = 3) or MASH livers (n = 3). (C) Normalized expression level of the stated gene in each mouse liver cell lineage. (D) UMAP showing annotation of 429 mouse liver mesenchymal single cells as portal fibroblasts, quiescent HSCs, activated HSCs, or mesothelia. (E) UMAP showing whether mesenchymal cells are from healthy (n = 3) or MASH livers (n = 3). (F) Normalized expression level of the stated gene in each mouse liver mesenchymal cell type. Abbreviations: MASH, metabolic dysfunction–associated steatohepatitis; MP, mononuclear phagocyte; scRNA-seq, single-cell RNA-sequencing.

The mechanisms by which FXR agonism mediates antifibrotic effects in MASH and the specific cellular target populations in vivo remain uncertain. To define candidate cell types, we interrogated liver single-cell RNA-sequencing (scRNA-seq) data using findings from the AMLN MASH model,^5^ with a focus on identifying cellular populations expressing both INT-767 target receptors (FXR (*Nr1h4*) and TGR5 (*Gpbar1*)) as well as the key ECM molecules (*Col1a1, Col3a1, Col4a1, Col4a2, Sparc, Fbn1, Lamb1*, and *Lamb2*) which were modulated by INT-767 treatment in viv*o* (Figure 1D). Analysis of all cell lineages demonstrated that mesenchymal cells expressed both *Nr1h4* and basement membrane genes (Figures 4A–C). No significant *Gpbar1* expression was detected in any cell lineage (data not shown). We then zoomed in on the mesenchymal cells, annotating 4 subpopulations (Figure 4D), including activated HSCs, which expand in MASH livers (Figure 4E). HSCs were the main *Nr1h4* expressing mesenchymal cell subpopulation and coexpressed fibrillar collagens and the specific basement membrane genes which were downregulated by INT-767 therapy (Figure 4F).

**FIGURE 5:**
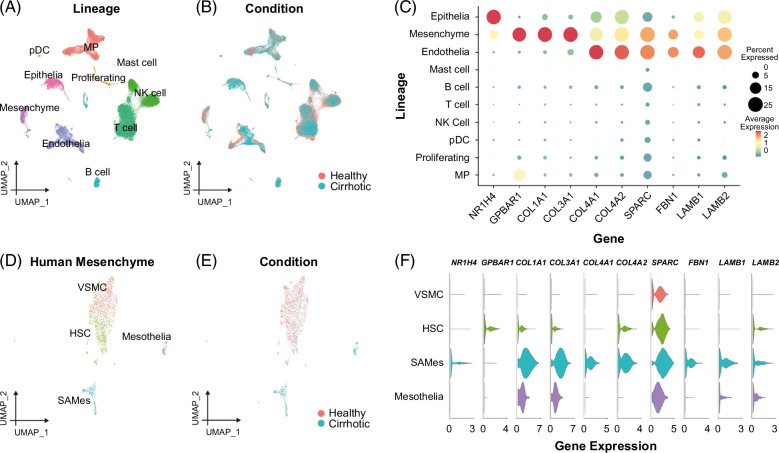
Human SAMes are a candidate target population for INT-767. (A) UMAP showing annotation of human liver scRNA-seq data (58,358 cells) by cell lineage (B) UMAP showing whether cells are from healthy (n = 5) or cirrhotic livers (n = 5). (C) Dot plot showing normalized expression level (color) and percentage of cells (dot size) expressing the stated gene in each cell lineage. (D) UMAP showing annotation of 2316 human liver mesenchymal single cells as VSMC, HSCs, mesothelia, or SAMes. (E) UMAP showing whether mesenchymal cells are from healthy (n = 5) or cirrhotic livers (n = 5). (F) Normalized expression level of the stated gene in each human liver mesenchymal cell type. Abbreviations: SAMes, scar-associated mesenchymal cells; scRNA-seq, single-cell RNA-sequencing; VSMC, vascular smooth muscle cells.

To assess whether human HSCs are also candidate cellular targets for the antifibrotic effects of FXR agonists, we analyzed scRNA-seq data from healthy and cirrhotic human liver tissue.^39^ Analysis of the human liver single-cell atlas demonstrated *NR1H4* and *GPBAR1* expression in both epithelial and mesenchymal cells (Figures 5A–C), but only mesenchymal cells coexpressed significant levels of fibrillar collagens and basement membrane molecules (Figure 5C). A distinct subpopulation of scar-associated mesenchymal cells (SAMes), most likely derived from the activation and differentiation of HSCs, populates the fibrotic niche in the fibrotic human liver and expresses high levels of fibrillar collagens.^39^ Targeted scRNA-seq analysis of human liver mesenchymal cells showed that human SAMes expand in cirrhotic livers and coexpress *NR1H4*, fibrillar collagens, and basement membrane molecules (Figures 5D–F), highlighting this population as a candidate cellular target for FXR agonists in diseased human liver. Hence, HSCs in both humans and mice coexpress NR1H4 and pathogenic basement membrane molecules.

### INT-767 attenuates basement membrane production by activated human HSCs in vitro

**FIGURE 6:**
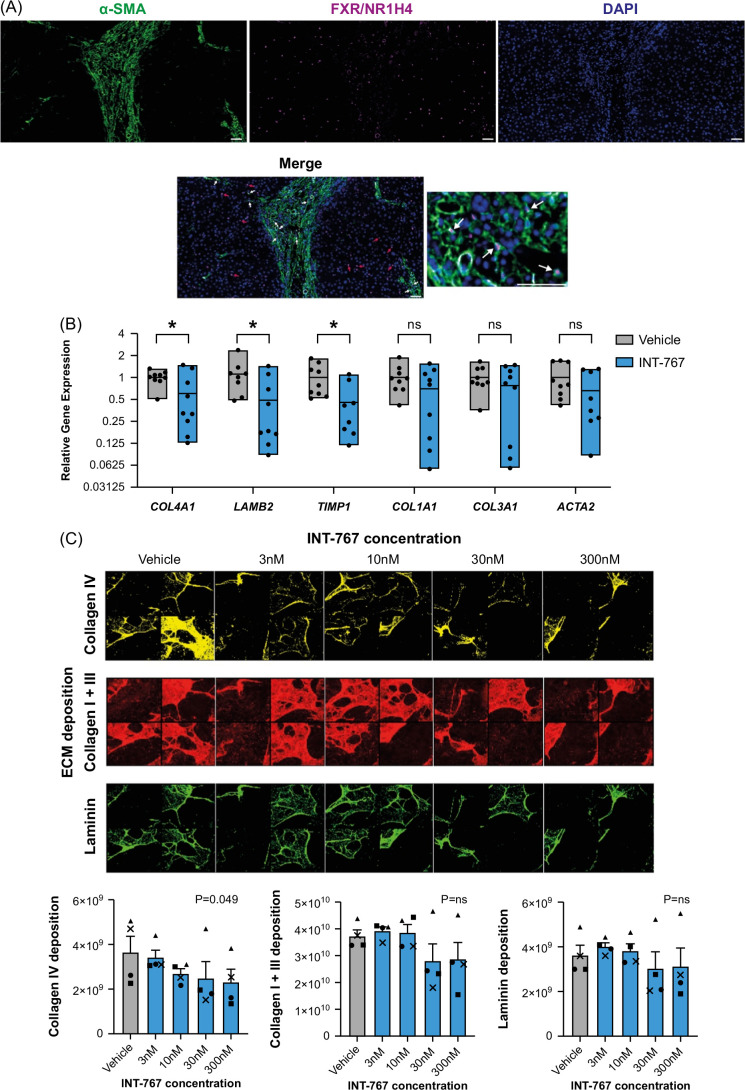
INT-767 regulates basement membrane production by HSCs. (A) Representative immunofluorescence images of human MASH liver tissue stained for NR1H4/FXR and a-SMA. Individual channel images and merged images are shown. White arrows indicating NR1H4^+^ α-SMA^+^ cells; red arrows indicating NR1H4^+^ α-SMA^−^ cells. Scale bar = 50 μm. (B) Culture-activated primary human HSCs treated with INT-767 (n = 8–9) or vehicle control (n = 8–9) from 3 independent experiments. Expression of stated gene quantified by qPCR and expressed relative to mean gene expression for vehicle control. Levels of significance: *p < 0.05, ns (Col1a1) *p* = 0.22, ns (Col3a1) *p* = 0.34, ns (Acta2) *p* = 0.20 (unpaired *t* test). (C) Deposition of ECM components collagen IV, collagen I + III, and laminin by culture-activated primary human HSCs assessed by immunofluorescent staining in response to stated concentration of INT-767 or vehicle (DMSO) control. Exemplar images from 4 wells at different INT-767 concentrations shown. Data expressed as average total positive pixels per well. Analysis from 4 independent experiments; symbols indicate separate experiments. Levels of significance: ns (collagen I + III) *p* = 0.58, ns (laminin) *p* = 0.48 (Friedman test). Abbreviations: ECM, extracellular matrix; MASH, metabolic dysfunction–associated steatohepatitis.

To confirm that activated HSCs represent the likely ECM-expressing cellular target of INT-767 in vivo, we performed immunofluorescence staining, demonstrating that human SAMes express NR1H4 within the fibrotic niche (Figure 6A; white arrows). Notably, in keeping with our scRNA-seq data, NR1H4 nuclear staining was not exclusive to SAMes (Figure 6A; red arrows). We then evaluated the direct effects of INT-767 on activated human HSCs in vitro. qPCR confirmed the expression of *NR1H4*, but not *TGR5*, in culture-activated primary human HSCs (data not shown). Treatment of culture-activated human HSCs with INT-767 resulted in significant downregulation of basement membrane genes *COL4A1* and *LAMB2* with a trend to reduction of other profibrogenic genes (Figure 6B). At the protein level, an ECM deposition assay demonstrated a dose-dependent reduction in collagen IV in response to INT-767. No significant difference was observed for collagen I and III or laminin, highlighting the predominant effects of INT-767 on collagen IV deposition by HSCs. No effects of INT-767 were detected on HSC proliferation (Supplemental Figure S5C, http://links.lww.com/HC9/B86). These findings demonstrate that INT-767 has a direct effect on activated HSCs to modulate ECM and, specifically, basement membrane expression.

## DISCUSSION

In this study, we characterized the antifibrotic effects of INT-767, an orally administered dual agonist of FXR and TGR5, demonstrating a prominent reduction in hepatic basement membrane molecule production in response to INT-767 and identifying activated HSCs as the cellular target of INT-767 in MASH. FXR agonists are already under investigation as antifibrotic agents in clinical trials in patients with MASH,^40^ but the mechanism of action and target ECM components have remained unclear. Our findings have important implications for the understanding of the functional sequelae of FXR agonism in vivo, highlighting a reduction in basement membrane ECM expression as a potentially more refined biological readout of drug efficacy, which could be explored further in a post hoc analysis of biopsy and/or serum samples from completed trials.

INT-767, like OCA, is derived from the primary human bile acid chenodeoxycholic acid, the natural endogenous FXR agonist. However, INT-767 is a more potent FXR agonist than OCA and, therefore, could demonstrate greater efficacy in human liver disease. In a comparative preclinical analysis, both OCA and INT-767 significantly improved all histological parameters relative to control, but INT-767 outperformed OCA at both matched and potency-adjusted doses.^21^ To date, INT-767 has completed phase 1 clinical development, the goal of which was to assess safety and pharmacokinetics in healthy volunteers, but it has not been investigated in patients with MASH and fibrosis. Here, using a well-validated *ob/ob* mouse model of AMLN diet-induced and biopsy-proven MASH and hepatic fibrosis, the most striking effect of INT-767 was modulation of basement membrane ECM production, assessed by serum markers, tissue gene expression, and immunohistochemistry analyses.

Most preclinical pharmacology studies in MASH and hepatic fibrosis have focused on fibrillar collagens (type I and type III) but have overlooked the basement membrane ECM, which is comprised of type IV collagen, laminin, nidogen, and sulfated proteoglycans. Key roles of the basement membrane ECM include compartmentalization of tissues, providing sites for cell adhesion, and serving as a source of signaling cues that regulate cellular proliferation, migration, and differentiation. In fibrosis, the composition and function of the basement membrane are critically altered. In a recent multi-transcriptome analysis, COL4A2 and LAMB1 were identified as prioritized genes specifically associated with liver fibrosis progression, independent of etiology.^38^ Specifically in patients with MASH, type IV collagen may represent a useful surrogate measure of disease severity.^41^ Serum markers of type IV collagen have also been shown to predict fibrosis recurrence and differentiate between intermediate and fast progressors in patients who received a liver transplant.^42^ We have profiled liver gene expression in a multicentre human MASLD cohort, showing that basement membrane constituents were positively associated with both fibrosis stage and adverse clinical outcomes and demonstrating that these molecules contribute to the ECM composition of the fibrotic niche. Interestingly, FXR regulon activity showed an inverse correlation with basement membrane components and was lower in patients with worse clinical outcomes, emphasizing the potential benefits of FXR agonism as a therapeutic strategy in MASLD. Going forward, rather than only focusing on fibrillar collagens, a more nuanced assessment of specific ECM components, including basement membrane proteins, could adequately characterize and quantify the antifibrotic effect of FXR agonists and other candidate therapeutic interventions.

In the present AMLN *ob/ob*-MASH mouse model, a serum marker of type IV procollagen processing (PRO-C4) tracked tissue levels of fibrillar and basement membrane collagens over time. Reduction in PRO-C4 observed in INT-767-treated animals correlated with both type I collagen and type IV collagen tissue staining, pointing to its utility as a potential drug response biomarker. Similarly, circulating levels of type IV collagen markers have been shown to correlate with hepatic tissue levels of type IV collagen in patients with alcohol-associated liver disease and chronic hepatitis C^43^ and to decrease following successful therapy.^44^^,^^45^ A limitation of the current study is the inclusion of a single animal model, although this model demonstrates good clinical translatability in head-to-head comparisons with patients with MASH,^27^ is frequently used by BioPharma for preclinical evaluation of MASH pharmacotherapies, and was employed here in both progression and regression modes. In addition, the model incorporated a baseline (pretreatment) stratification liver biopsy with robust group sizes and was supported by longitudinal serum biomarker analyses.

To confirm the cellular target of FXR agonism in human chronic liver disease (including MASH), we used scRNA-seq to identify fibrogenic SAMes cells as the predominant cell type expressing both FXR and the ECM molecules shown to be modulated by INT-767, and then spatially mapped these FXR^+^ myofibroblasts to the fibrotic niche. We confirmed these findings using murine scRNA-seq data, defining activated HSCs as the most likely target population to mediate the antifibrotic effects of INT-767 in vivo. We went on to show the direct modulatory effects of INT-767 on activated primary human HSCs in vitro, resulting in the downregulation of basement membrane expression. This extends recent observations showing that both OCA and INT-767 induced a dose-dependent reduction in free fatty acid–induced type I collagen deposition in a MASH co-culture model using human HSCs and hepatocytes,^20^ and highlights activated HSCs as a likely target population for FXR agonists in the diseased human liver. While we have focussed on HSCs in this study, our scRNA-seq data and previous literature demonstrate that other cells in the liver, including biliary epithelial cells and sinusoidal endothelial cells, also express FXR. In addition to the direct effects of INT-767 on HSCs, it is therefore possible that the effects of INT-767 on other hepatic cell populations may modulate HSC phenotype and function in an indirect manner. Future studies could employ scRNA-seq following INT-767 treatment to study transcriptional responses in different cell types and model changes in ligand-receptor interactions between these populations.

While the beneficial effect of INT-767 over OCA in modulating MASH in vivo is thought to reflect greater potency of FXR target gene modulation,^21^ INT-767 also acts as a TGR5 agonist.^19^ Previous comparisons between INT-767 and INT-777 (a specific TGR5 agonist) *in vivo* have shown that only INT-767 reduced liver fibrosis in MASH^22^ and biliary injury^23^ models, while TGR5 activation has recently been suggested to be profibrogenic in MASH.^46^ Furthermore, in vivo antifibrotic effects of INT-767 in murine MASH were abrogated in FXR-deficient animals, whereas TGR5 deficiency had no impact on INT-767 efficacy.^22^ TGR5 has also been shown to modulate lipid metabolism,^47^ have anti-inflammatory effects in the mouse liver,^48^ and protect against biliary injury.^49^ Future work will be required to dissect the relative contribution of TGR5 agonism to basement membrane turnover effects of INT-767 in MASH. In addition, INT-767 has been shown to alter the bile acid pool in mice in vivo, modulating hepatic pathways involved in bile acid synthesis, excretion, resorption, and efflux and resulting in reduced hydrophobicity of bile acids.^22^^,^^32^ Similar to these publications, our liver RNA-seq data demonstrates reduced expression of classical bile acid synthesis enzymes (Cyp7a1 and Cyp8b1), increased expression of the bile acid transporter for excretion (Bsep/Abcb11), decreased expression of the liver bile acid transporters for resorption (Oatp/Slco1a4), and increased expression of bile acid transporter for efflux to the blood circulation (OstB/Slc51b) in response to INT-767 treatment. We did not measure bile acid composition directly in this study, and it is possible that altered bile acid composition in response to INT-767 may directly regulate ECM and basement membrane production, something which should be mechanistically explored in future studies, ideally using human cells and tissue where possible. INT-767 also has functional effects at other sites, such as adipose tissue,^24^ which may play a role in MASH pathogenesis. More detailed future studies of the systemic effects of FXR agonism and how these might regulate the liver phenotype should also be considered.

In summary, INT-767 showed antifibrotic activity in preclinical in vivo and in vitro models, with a pronounced effect on basement membrane proteins, although its efficacy in human patients with MASH has not yet been determined. Our findings highlight the importance of basement membrane ECM as a pathological feature of MASH-related fibrosis and its potential as a therapeutic target and a source of dynamic companion diagnostic biomarkers.

## Supplementary Material

## References

[1] Younossi ZM Blissett D Blissett R Henry L Stepanova M Younossi Y. The economic and clinical burden of nonalcoholic fatty liver disease in the United States and Europe. Hepatology. 2016;64:1577–1586. 27543837 10.1002/hep.28785

[2] Harrison SA Bedossa P Guy CD Schattenberg JM Loomba R Taub R. A phase 3, randomized, controlled trial of resmetirom in NASH with liver fibrosis. N Engl J Med. 2024;390:497–509. 38324483 10.1056/NEJMoa2309000

[3] Sanyal AJ Van Natta ML Clark J Neuschwander-Tetri BA Diehl A Dasarathy S. Prospective study of outcomes in adults with nonalcoholic fatty liver disease. N Engl J Med. 2021;385:1559–1569. 34670043 10.1056/NEJMoa2029349 PMC8881985

[4] Trivedi P Wang S Friedman SL. The power of plasticity—Metabolic regulation of hepatic stellate cells. Cell Metab. 2021;33:242–257. 33232666 10.1016/j.cmet.2020.10.026 PMC7858232

[5] Xiong X Kuang H Ansari S Liu T Gong J Wang S. Landscape of intercellular crosstalk in healthy and NASH liver revealed by single-cell secretome gene analysis. Mol Cell. 2019;75:644–660.e5. 31398325 10.1016/j.molcel.2019.07.028 PMC7262680

[6] Karsdal MA Nielsen SH Leeming DJ Langholm LL Nielsen MJ Manon-Jensen T. The good and the bad collagens of fibrosis—Their role in signaling and organ function. Adv Drug Deliv Rev. 2017;121:43–56. 28736303 10.1016/j.addr.2017.07.014

[7] Wu Y Cao Y Xu K Zhu Y Qiao Y Wu Y. Dynamically remodeled hepatic extracellular matrix predicts prognosis of early-stage cirrhosis. Cell Death Dis. 2021;12:163. 33558482 10.1038/s41419-021-03443-y PMC7870969

[8] Nielsen MJ Karsdal MA Kazankov K Grønbaek H Krag A Leeming DJ. Fibrosis is not just fibrosis—Basement membrane modelling and collagen metabolism differs between hepatitis B- and C-induced injury. Aliment Pharmacol Ther. 2016;44:1242–1252. 27696451 10.1111/apt.13819

[9] Sørensen MD Thiele M Krag A Daniels SJ Leeming DJ Karsdal M. Stage-dependent expression of fibrogenic markers in alcohol-related liver disease. Pathol Res Pract. 2022;231:153798. 35180651 10.1016/j.prp.2022.153798

[10] Mak KM Mei R. Basement membrane type IV collagen and laminin: An overview of their biology and value as fibrosis biomarkers of liver disease. Anat Rec. 2017;300:1371–1390. 10.1002/ar.23567 28187500

[11] Stefano JT Guedes LV de Souza AAA Vanni DS Alves VAF Carrilho FJ. Usefulness of collagen type IV in the detection of significant liver fibrosis in nonalcoholic fatty liver disease. Ann Hepatol. 2021;20:100253. 32949785 10.1016/j.aohep.2020.08.070

[12] Ishiba H Sumida Y Seko Y Tanaka S Yoneda M Hyogo H. Type IV collagen 7S is the most accurate test for identifying advanced fibrosis in NAFLD with type 2 diabetes. Hepatol Commun. 2021;5:559–572. 33860115 10.1002/hep4.1637 PMC8034577

[13] Younossi ZM Ratziu V Loomba R Rinella M Anstee QM Goodman Z. Obeticholic acid for the treatment of non-alcoholic steatohepatitis: Interim analysis from a multicentre, randomised, placebo-controlled phase 3 trial. The Lancet. 2019;394:2184–2196. 10.1016/S0140-6736(19)33041-7 31813633

[14] Chianelli D Rucker PV Roland J Tully DC Nelson J Liu X. Nidufexor (LMB763), a novel FXR modulator for the treatment of nonalcoholic steatohepatitis. J Med Chem. 2020;63:3868–3880. 31940200 10.1021/acs.jmedchem.9b01621

[15] Fiorucci S Antonelli E Rizzo G Renga B Mencarelli A Riccardi L. The nuclear receptor SHP mediates inhibition of hepatic stellate cells by FXR and protects against liver fibrosis. Gastroenterology. 2004;127:1497–1512. 15521018 10.1053/j.gastro.2004.08.001

[16] Fiorucci S Rizzo G Antonelli E Renga B Mencarelli A Riccardi L. Cross-talk between farnesoid-X-receptor (FXR) and peroxisome proliferator-activated receptor gamma contributes to the antifibrotic activity of FXR ligands in rodent models of liver cirrhosis. J Pharmacol Exp Ther. 2005;315:58–68. 15980055 10.1124/jpet.105.085597

[17] Fickert P Fuchsbichler A Moustafa T Wagner M Zollner G Halilbasic E. Farnesoid X receptor critically determines the fibrotic response in mice but is expressed to a low extent in human hepatic stellate cells and periductal myofibroblasts. Am J Pathol. 2009;175:2392–2405. 19910507 10.2353/ajpath.2009.090114 PMC2789609

[18] Verbeke L Mannaerts I Schierwagen R Govaere O Klein S Vander Elst I. FXR agonist obeticholic acid reduces hepatic inflammation and fibrosis in a rat model of toxic cirrhosis. Sci Rep. 2016;6:33453. 27634375 10.1038/srep33453 PMC5025787

[19] Rizzo G Passeri D De Franco F Ciaccioli G Donadio L Rizzo G. Functional characterization of the semisynthetic bile acid derivative INT-767, a dual farnesoid X receptor and TGR5 agonist. Mol Pharmacol. 2010;78:617–630. 20631053 10.1124/mol.110.064501 PMC2981390

[20] Anfuso B Tiribelli C Adorini L Rosso N. Obeticholic acid and INT-767 modulate collagen deposition in a NASH in vitro model. Sci Rep. 2020;10:1699. 32015483 10.1038/s41598-020-58562-x PMC6997404

[21] Roth JD Feigh M Veidal SS Fensholdt LKD Rigbolt KT Hansen HH. INT-767 improves histopathological features in a diet-induced ob/ob mouse model of biopsy-confirmed non-alcoholic steatohepatitis. World J Gastroenterol. 2018;24:195–210. 29375205 10.3748/wjg.v24.i2.195 PMC5768938

[22] Wang XX Xie C Libby AE Ranjit S Levi J Myakala K. The role of FXR and TGR5 in reversing and preventing progression of Western diet-induced hepatic steatosis, inflammation, and fibrosis in mice. J Biol Chem. 2022;298:102530. 36209823 10.1016/j.jbc.2022.102530 PMC9638804

[23] Baghdasaryan A Claudel T Gumhold J Silbert D Adorini L Roda A. Dual farnesoid X receptor/TGR5 agonist INT-767 reduces liver injury in the Mdr2 −/− ( Abcb4 −/− ) mouse cholangiopathy model by promoting biliary HCO 3− output. Hepatology. 2011;54:1303–1312. 22006858 10.1002/hep.24537 PMC3744065

[24] Comeglio P Cellai I Mello T Filippi S Maneschi E Corcetto F. INT-767 prevents NASH and promotes visceral fat brown adipogenesis and mitochondrial function. J Endocrinol. 2018;238:107–127. 29945982 10.1530/JOE-17-0557

[25] Jadhav K Xu Y Xu Y Li Y Xu J Zhu Y. Reversal of metabolic disorders by pharmacological activation of bile acid receptors TGR5 and FXR. Mol Metab. 2018;9:131–140. 29361497 10.1016/j.molmet.2018.01.005 PMC5870099

[26] Baandrup Kristiansen MN Veidal SS Christoffersen C Feigh M Vrang N Roth JD. Validity of biopsy-based drug effects in a diet-induced obese mouse model of biopsy-confirmed NASH. BMC Gastroenterol. 2019;19:228. 31883514 10.1186/s12876-019-1149-z PMC6935483

[27] Hansen HH Ægidius HM Oró D Evers SS Heebøll S Eriksen PL. Human translatability of the GAN diet-induced obese mouse model of non-alcoholic steatohepatitis. BMC Gastroenterol. 2020;20:210. 32631250 10.1186/s12876-020-01356-2 PMC7336447

[28] Kleiner DE Brunt EM Van Natta M Behling C Contos MJ Cummings OW. Design and validation of a histological scoring system for nonalcoholic fatty liver disease. Hepatology. 2005;41:1313–1321. 15915461 10.1002/hep.20701

[29] Kendall TJ Jimenez-Ramos M Turner F Ramachandran P Minnier J McColgan MD. An integrated gene-to-outcome multimodal database for metabolic dysfunction-associated steatotic liver disease. Nat Med. 2023;29:2939–2953. 37903863 10.1038/s41591-023-02602-2 PMC10667096

[30] Ijssennagger N Janssen AWF Milona A Ramos Pittol JM Hollman DAA Mokry M. Gene expression profiling in human precision cut liver slices in response to the FXR agonist obeticholic acid. J Hepatol. 2016;64:1158–1166. 26812075 10.1016/j.jhep.2016.01.016

[31] Roth JD Veidal SS Fensholdt LKD Rigbolt KTG Papazyan R Nielsen JC. Combined obeticholic acid and elafibranor treatment promotes additive liver histological improvements in a diet-induced ob/ob mouse model of biopsy-confirmed NASH. Sci Rep. 2019;9:9046. 31227742 10.1038/s41598-019-45178-z PMC6588626

[32] Pathak P Liu H Boehme S Xie C Krausz KW Gonzalez F. Farnesoid X receptor induces Takeda G-protein receptor 5 cross-talk to regulate bile acid synthesis and hepatic metabolism. J Biol Chem. 2017;292:11055–11069. 28478385 10.1074/jbc.M117.784322 PMC5491788

[33] Lambert G Amar MJA Guo G Brewer HBJ Gonzalez FJ Sinal CJ. The farnesoid X-receptor is an essential regulator of cholesterol homeostasis. J Biol Chem. 2003;278:2563–2570. 12421815 10.1074/jbc.M209525200

[34] Villesen IF Daniels SJ Leeming DJ Karsdal MA Nielsen MJ. Review article: The signalling and functional role of the extracellular matrix in the development of liver fibrosis. Aliment Pharmacol Ther. 2020;52:85–97. 32419162 10.1111/apt.15773

[35] Leeming DJ Nielsen MJ Dai Y Veidal SS Vassiliadis E Zhang C. Enzyme-linked immunosorbent serum assay specific for the 7S domain of collagen type IV (P4NP 7S): A marker related to the extracellular matrix remodeling during liver fibrogenesis. Hepatol Res. 2012;42:482–493. 22221767 10.1111/j.1872-034X.2011.00946.x

[36] Luo Y Oseini A Gagnon R Charles ED Sidik K Vincent R. An evaluation of the collagen fragments related to fibrogenesis and fibrolysis in nonalcoholic steatohepatitis. Sci Rep. 2018;8:12414. 30120271 10.1038/s41598-018-30457-y PMC6098042

[37] Sand JM Larsen L Hogaboam C Martinez F Han M Røssel Larsen M. MMP mediated degradation of type IV collagen alpha 1 and alpha 3 chains reflects basement membrane remodeling in experimental and clinical fibrosis—Validation of two novel biomarker assays. PLoS One. 2013;8:e84934. 24376856 10.1371/journal.pone.0084934 PMC3871599

[38] Chen W Wu X Yan X Xu A Yang A You H. Multitranscriptome analyses reveal prioritized genes specifically associated with liver fibrosis progression independent of etiology. Am J Physiol-Gastrointest Liver Physiol. 2019;316:G744–G754. 30920297 10.1152/ajpgi.00339.2018

[39] Ramachandran P Dobie R Wilson-Kanamori JR Dora EF Henderson BEP Luu NT. Resolving the fibrotic niche of human liver cirrhosis at single-cell level. Nature. 2019;575:512–518. 31597160 10.1038/s41586-019-1631-3 PMC6876711

[40] Harrison SA Bashir MR Lee K-J Shim-Lopez J Lee J Wagner B. A structurally optimized FXR agonist, MET409, reduced liver fat content over 12 weeks in patients with non-alcoholic steatohepatitis. J Hepatol. 2021;75:25–33. 33581174 10.1016/j.jhep.2021.01.047

[41] Mizuno M Shima T Oya H Mitsumoto Y Mizuno C Isoda S. Classification of patients with non-alcoholic fatty liver disease using rapid immunoassay of serum type IV collagen compared with liver histology and other fibrosis markers. Hepatol Res. 2017;47:216–225. 26997642 10.1111/hepr.12710

[42] Nielsen MJ Villesen IF Gudmann NS Leeming DJ Krag A Karsdal MA. Serum markers of type III and IV procollagen processing predict recurrence of fibrosis in liver transplanted patients. Sci Rep. 2019;9:14857. 31619707 10.1038/s41598-019-51394-4 PMC6796007

[43] Laursen TL Villesen IF Leeming DJ Karsdal MA Sølund C Tarp B. Altered balance between collagen formation and degradation after successful direct-acting antiviral therapy of chronic hepatitis C. J Viral Hepat. 2021;28:236–244. 33058390 10.1111/jvh.13416

[44] Yabu K Kiyosawa K Mori H Matsumoto A Yoshizawa K Tanaka E. Serum collagen type IV for the assessment of fibrosis and resistance to interferon therapy in chronic hepatitis C. Scand J Gastroenterol. 1994;29:474–479. 7518613 10.3109/00365529409096841

[45] Tsutsumi M Takase S Urashima S Ueshima Y Kawahara H Takada A. Serum markers for hepatic fibrosis in alcoholic liver disease: Which is the best marker, type III procollagen, type IV collagen, laminin, tissue inhibitor of metalloproteinase, or prolyl hydroxylase? Alcohol Clin Exp Res. 1996;20:1512–1517. 8986196 10.1111/j.1530-0277.1996.tb01692.x

[46] Xie G Jiang R Wang X Liu P Zhao A Wu Y. Conjugated secondary 12α-hydroxylated bile acids promote liver fibrogenesis. EBioMedicine. 2021;66:103290. 33752128 10.1016/j.ebiom.2021.103290 PMC8010625

[47] Donepudi AC Boehme S Li F Chiang JYL. G-protein-coupled bile acid receptor plays a key role in bile acid metabolism and fasting-induced hepatic steatosis in mice. Hepatology. 2017;65:813–827. 27351453 10.1002/hep.28707 PMC5195921

[48] Shi Y Su W Zhang L Shi C Zhou J Wang P. TGR5 regulates macrophage inflammation in nonalcoholic steatohepatitis by modulating NLRP3 inflammasome activation. Front Immunol. 2020;11:609060. 33692776 10.3389/fimmu.2020.609060 PMC7937818

[49] Reich M Spomer L Klindt C Fuchs K Stindt J Deutschmann K. Downregulation of TGR5 (GPBAR1) in biliary epithelial cells contributes to the pathogenesis of sclerosing cholangitis. J Hepatol. 2021;75:634–646. 33872692 10.1016/j.jhep.2021.03.029

