# Supplementary material for: Aberrant basement membrane production by HSCs in MASLD is attenuated by the bile acid analog INT-767
Source: Hepatol Commun. 2024 Nov 25;8(12):e0574. doi: 10.1097/HC9.0000000000000574 (PMC11596521; doi:10.1097/HC9.0000000000000574)
Supplement: Supplementary file 1 [file hc9-8-e0574-s001.docx]

# Supplementary Digital Content

**Title:** **Aberrant basement membrane production by hepatic stellate cells in MASLD is attenuated by the bile acid analog INT-767**

**Authors**: Prakash Ramachandran^1^*, Madara Brice^1^, Elena Sutherland^1^, Anna M Hoy^1^, Eleni Papachristoforou^1^, Li Jia^1^, Frances Turner^2^, Timothy J Kendall^1,3^, John A Marwick^1^, Neil O. Carragher^4^, Denise Oro^5^, Michael Feigh^5^, Diana J Leeming^6^, Mette J Nielsen^6^, Morten A Karsdal^6^, Nadine Hartmann^7^, Mary Erickson^7^, Luciano Adorini^7^, Jonathan D Roth^7^, Jonathan A Fallowfield^1^

**Affiliations**: ^1^Institute for Regeneration and Repair, University of Edinburgh, Edinburgh, United Kingdom; ^2^Edinburgh Genomics, University of Edinburgh, Edinburgh, United Kingdom; ^3^Edinburgh Pathology, University of Edinburgh, Edinburgh, United Kingdom; ^4^Cancer Research UK Scotland Centre, Institute of Genetics and Cancer, University of Edinburgh, Edinburgh, United Kingdom; ^5^Gubra, Hørsholm, Denmark; ^6^Nordic Bioscience, Herlev, Denmark; ^7^Intercept Pharmaceuticals Inc., San Diego, CA, United States.

***Corresponding author**: Prof Prakash Ramachandran, Centre for Inflammation Research, Institute for Regeneration and Repair, University of Edinburgh, Edinburgh BioQuarter, 4-5 Little France Drive, Edinburgh, EH16 4UU, United Kingdom.

Tel: +44(0)131 242 9164.

Email: [Prakash.Ramachandran@ed.ac.uk](mailto:Prakash.Ramachandran@ed.ac.uk)

## SDC, Materials and Methods

**Serum analysis**

Serum was analyzed from fasted mice at week -3, week 0, and after 4 and 8 weeks of treatment for PRO-C4 (a marker of type IV collagen formation) using an ELISA targeting an internal epitope in the 7S domain of type IV collagen(1) and C4M (a type IV collagen degradation marker)(2), both according to the manufacturer (Nordic Bioscience, Herlev, Denmark). Terminal blood samples from non-fasted mice were assayed for plasma concentrations of alanine aminotransferase (ALT), aspartate aminotransferase (AST), triglycerides (TG) and total cholesterol (TC).

***Gene Expression***

***Mouse liver bulk RNA-sequencing (RNA-seq)*:** Murine hepatic transcriptomic analysis was performed by RNA-seq on RNA extracts from terminal liver samples, as previously described(3). RNA-seq libraries were prepared with NeoPrep (Illumina Inc., San Diego, CA, US) using Illumina TruSeq stranded mRNA Library kit for NeoPrep (Illumina) and sequenced on the NextSeq 500 (Illumina) with NSQ 500 hi-Output KT v2 (75 CYS; Illumina). Reads were aligned to the GRCm38 v84 Ensembl Mus musculus genome using STAR(4) v.2.5.2a with default parameters. Differential gene expression analysis was performed with edgeR(5). Differentially expressed genes were grouped into 2 hierarchical clusters using dendextend(6). Functional annotation of differentially expressed genes was performed using the DAVID tool(7,8). Raw data has been deposited to the Gene Expression Omnibus (GEO) under accession number GSE275813.

***Human liver bulk RNA-seq*:** Unified transparent approval for the pan-Scotland SteatoSITE project(9) was provided by the West of Scotland Research Ethics Committee 4 (Reference: 20/WS/0002). RNA extraction was performed by the Genetics Core (Clinical Research Facility, Western General Hospital, Edinburgh, UK) from 4 x 10um curls of FFPE archival human NAFLD liver, using the QIAGEN miRNeasy FFPE Kit (Qiagen, Manchester, UK) according to the manufacturer’s instructions. Quality control was by Qubit to measure RNA yield (and any potential DNA contamination) and Agilent Bioanalyser (Agilent) to assess DV200. Samples with DV200 below 30% were not progressed for sequencing. Libraries were prepared at Precision Medicine Scotland-Innovation Centre (Glasgow, UK) using the low-input Takara Bio SMARTer Stranded Total RNA-Seq Kit v2 (Pico Input Mammalian; Takara Bio, Goteborg, Sweden). Sequencing data was generated by Edinburgh Genomics (Edinburgh, UK) using the Illumina NovaSeq 6000 platform (illumine, US). Sequenced reads were trimmed using Cutadapt(10) (version cutadapt 1.9.dev2) and trimmed for quality and adapter sequences at the 3’ end using a quality threshold of 30. Reads after trimming were required to have a minimum length of 50. Reads were aligned to the reference genome (build GRCh38) using STAR(4) (version 2.5.2b) specifying paired-end reads and the option --outSAMtype BAM Unsorted. All other parameters were left at default. Reads were assigned to features using featureCounts(11) (version 1.5.1) with a “gtf” file from Ensembl release 84 and using the parameter “-p -s 2 -Q 10“. Genes that did not have a least 0.1 counts per million (CPM) in at least 10 samples were removed from the analysis. Reads were normalized using the weighted trimmed mean of M-values method(12), passing ‘TMM’ as the method to the calcNormFactors method of edgeR. Differential analysis was carried out with edgeR(5) (version 3.20.9) using the quasi-likelihood (QL) F-test method. Differential gene set analysis was carried out with the ROAST method(13) from the Bioconductor package “Limma”(14) (version 3.34.9).

For correlation between NR1H4 regulon activity and fibrotic gene expression and time-to-event analysis, biopsy cases with available RNAseq from the SteatoSITE multimodal database(9) were used (R 4.3.3). Normalized counts for target genes were available as part of the dataset, and the NR1H4 regulon activity profile was generated as previously described(9). Decompensation events in the clinical data extract were defined by a combination of ICD codes and UK OPCS-4 codes identifying procedures relating to cirrhosis-related hospital admissions activity. Decompensation event analysis was only undertaken on biopsy cases for which the first decompensation-related coding was present in the clinical data extract after the biopsy date and analysis was undertaken using death as a competing risk. The cutpoint for gene or regulon activity was calculated using surv_cutpoint() from the ‘survminer’ package, applying maximally selected rank statistics of the ‘maxstat’ package with a minimum proportion of 0.25. Kaplan–Meier estimator curves of all-cause mortality or decompensation events for assigned high/low risk groups were compared by log-rank testing in ‘survminer’. Correlation between gene expression/regulon activity in the complete set of n=663 SteatoSITE samples with available RNAseq used Spearman's method and plotting was undertaken in 'rstatix'.

Human hepatic bulk RNA-seq data are deposited in the European Nucleotide Archive (<https://www.ebi.ac.uk/ena>; study accession number: [PRJEB58625](https://www.ebi.ac.uk/ena/data/view/PRJEB58625)), as described in the original publication(9).

***Human single-cell RNA-seq (scRNA-seq)*:** Previously published scRNAseq data following QC, unsupervised clustering, and annotation(15), was downloaded from a publicly available server for analysis (<https://doi.org/10.7488/ds/2637>). All analysis was performed in the Seurat R package (version 4.0.5). In total, 58,358 human cells from 5 healthy and 5 cirrhotic livers were analyzed(15). Data was analyzed using the built-in functions of Seurat to normalize the cells per 10,000 transcripts and then log(x+1) transformed allowing the resulting values to be scaled. Cells from all livers were combined for downstream analysis using the integration algorithm Harmony which accounts for multiple experimental and biological factors(16). Finally, the Uniform Approximation and Projection Method (UMAP) was used to construct a data visualization graph using 1 to 50 principal components as determined by the dataset variability shown in principal component analysis (PCA). Cells from these patients which were annotated as mesenchyme based on the original published analyses were then isolated for re-analysis using the same analysis workflow. To identify how the expression of genes of interest differ between cell lineages or the mesenchymal cells previously annotated as vascular smooth muscle cells (VSMC), hepatic stellate cells (HSC), mesothelia or scar-associated mesenchyme (SAMes) in healthy and cirrhotic liver tissue, UMAP visualizations, violin plots and dot plots were generated using Seurat functions in conjunction with the ggplot2 package.

***Mouse single-cell RNA-seq (scRNA-seq)*:** Mouse liver non-parenchymal cell scRNA-seq data from AMLN diet-induced MASH (n=3) and healthy control tissue (n=3) generated via the 10X Genomics platform, were available as processed gene matrices and obtained from GEO (GSE129516)(17). A total of 33,168 non-parenchymal liver cells from the 6 mice were imported and analyzed in Seurat R package (version 4.0.3). The merged Seurat object was normalized and scaled as described for human scRNA-seq analysis. Next, sample integration was carried out using the Harmony (version 1.0) package using default parameters(16). UMAP was given the top 50 harmony dimensions as an input and visualized at a clustering resolution of 1.4. Cluster marker genes were defined using the Seurat function FindMarkers with default parameters and clusters were annotated using previously described lineage marker genes(15,17). Cells annotated as mesenchymal cells were subsequently isolated and processed again through the pipeline described above without the Harmony integration step. A total of 429 mesenchymal cells were visualized using UMAP with the top 40 dimensions and clustering resolution of 0.15. Mesenchymal cell cluster marker genes were defined as above and clusters annotated as quiescent HSC, activated HSC, portal fibroblasts and mesothelial cells based on previously published marker genes(15,18). Expression of genes of interest were visualized both in the non-parenchymal mouse liver cells and mesenchymal cell subset with violin plots, using Seurat functions in conjunction with the ggplot2 package.

**qPCR**: RNA was isolated from primary human HSC using the RNeasy Plus Micro Kit (Qiagen, UK). Quantity of RNA was analzsed using a Qubit 3.0 Fluorometer (Life Technologies, ThermoFisher Scientific). cDNA synthesis was performed using QuantiTect Reverse Transcription Kit (Qiagen). Real-time quantitative PCR (qRT-PCR) was performed using PowerUp SYBR Green Master Mix (Applied Biosystems, ThermoFisher Scientific). Samples were amplified on an ABI 7900HT FAST PCR system (Applied Biosystems, ThermoFisher) and normalized against GAPDH. QuantiTect Primer Assays (#249900; Qiagen) were used for all genes (Supplementary Table 2). Data was analyzed using ThermoFisher Connect cloud software (ThermoFisher Scientific). Relative expression was determined by the comparative Ct method (2^-ΔΔCt^).

**Cell culture**

Primary human HSCs (#P10653; Innoprot, Bizkaia, Spain) were plated in T75 flasks pre-coated with 0.01% poly-L-lysine (#P4832; Sigma-Aldrich). Cells were initially cultured (37°C, 5% CO_2_) in SteCM growth medium (#5301; ScienCell Research Laboratories, Carlsbad, CA, US). After reaching 80% confluency, cells were trypsinized and plated at 100,000 cells/well in 12-well plates with standard HSC medium (Dulbecco’s Modified Eagle Medium (#21969-035; Gibco, ThermoFisher, UK) supplemented with 20 μM HEPES (#H3375; Sigma-Aldrich), 2 mM L-Glutamine (#25030-024; Gibco), 1% penicillin-streptomycin (#15140-122; Gibco) and 2% fetal bovine serum (#10270; Gibco) and allowed to adhere for 24 h. The cells were serum starved in serum-free HSC medium for 24 h before the start of experiments. For the qPCR, cells were incubated for 24 h with HSC media containing vehicle control (DMSO) or 10 μM of INT-767. All experiments were performed using cells between passage 3 and 5.

**Hepatic stellate cell ECM deposition and proliferation assay**

Human HSC proliferation(15) and ECM deposition(19) assays were performed as previously described. Briefly, human HSCs (#5300; ScienCell) were resuspended in treatment media (25,000 per mL) and seeded into 384-well plates (#781091; Greiner Bio-One, Gloucestershire, UK) at 25μL per well. For proliferation assay, serial dilutions of INT-767 were performed using treatment media containing DMSO (#D12345; Molecular Probes, ThermoFisher) at 1 in 1000 dilution, TGFb (#7754-BH; R&D Systems, Minneapolis, MN, US) at 10ng/mL and Nuclight Rapid Red (#4717; Sartorius, Goettingen, Germany) at 1 in 500 dilution (all concentrations at 2x final assay concentration). INT-767 (concentration as stated) or DMSO control were then added to the plates at 25μL per well and the plates pulse spun at 200g for 5 sec. The plates were then imaged using an Incucyte Zoom live cell imaging system (Sartorius) humidified at 37 °C with 5% CO_2_ with imaging every 3 h for 5 days using the 10X optic. The rate of proliferation was quantified by nuclei counts using Incucyte propriety image analysis software (version 2018A). Data are expressed as the percentage change in nuclei number from baseline versus time (h), calculated in GraphPad Prism. For ECM deposition assay, cells were lysed using an ammonia-based lysis media (0.25 M NH_4_OH, 25 mM Tris) for 30 min at 37 °C, followed by washing three times with phosphate-buffered saline (PBS). The ECM was fixed using ice-cold methanol for 20 min at –20 °C, followed by washing three times with PBS. Assay plates were then blocked followed by ECM staining and imaging as previously described in detail(19). The intensity of the fluorescent antibody labelling for each individual ECM channel was measured and aggregated using the SUM function. Mean intensity across 4 technical replicates per treatment condition were calculated. Experiments were repeated on 4 independent occasions.

## SDC, Figure 1

**
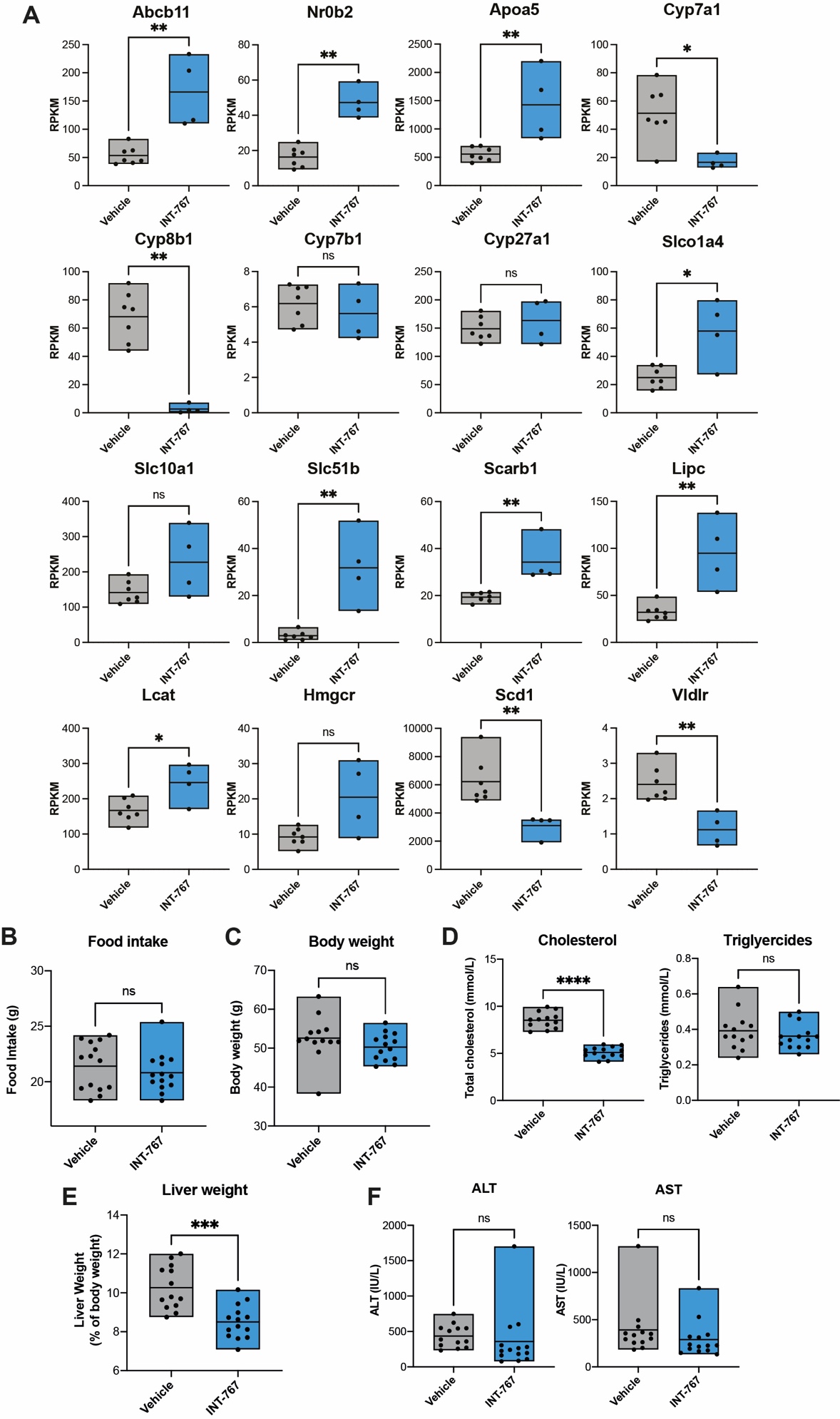
**

**Supplementary Fig. 1. INT-767 upregulates FXR target gene and does not modulate hepatocyte damage in AMLN *ob/ob*-MASH mice.** (A) Comparison of expression levels of stated FXR target gene between vehicle (n=7) and INT-767 (n=4) treated mice using RNA-seq data. RPKM: Reads per kilobase of transcript per million mapped reads. Levels of significance: **(Abcb11) p=0.0061, **(Nr0b2) p=0.0061, **(Apoa5) p=0.0061, *(Cyp7a1) p=0.0121, **(Cyp8b1) p=0.0061, n.s. (Cyp7b1) p=0.527, n.s. (Cyp27a1) p=0.649, *(Slco1a4) p=0.0424, n.s. (Slc10a1) p=0.109, **(Slc51b) p=0.0061, **(Scarb1) p=0.0061, **(Lipc) p=0.0061, *(Lcat) p=0.0424, n.s. (Hmgcr) p=0.0727, **(Scd1) p=0.0061, **(Vldlr) p=0.0061 (Mann-Whitney test). (B) Cumulative food intake (g) in vehicle (n=14) and INT-767 (n=14) treated animals during ongoing injury. Levels of significance: n.s. p=0.433 (unpaired *t*-test). (C) Body weight (g) in vehicle (n=13) and INT-767 (n=14) treated animals after ongoing injury. Levels of significance: n.s. p=0.214 (unpaired *t*-test). (D) Serum total cholesterol and triglycerides in vehicle (n=13) and INT-767 (n=14) treated animals after ongoing injury. Levels of significance: **** p<0.0001, n.s. p=0.384 (unpaired *t*-test). (E) Liver weight (as a % of body weight) in vehicle (n=13) and INT-767 (n=14) treated animals after ongoing injury. Levels of significance: *** p=0.0001 (unpaired *t*-test). (F) Serum ALT and AST in vehicle (n=13) and INT-767 (n=14) treated animals after ongoing injury. Levels of significance: n.s. (ALT) p=0.529, n.s. (AST) p=0.269 (unpaired *t*-test).

## SDC, Figure 2

**
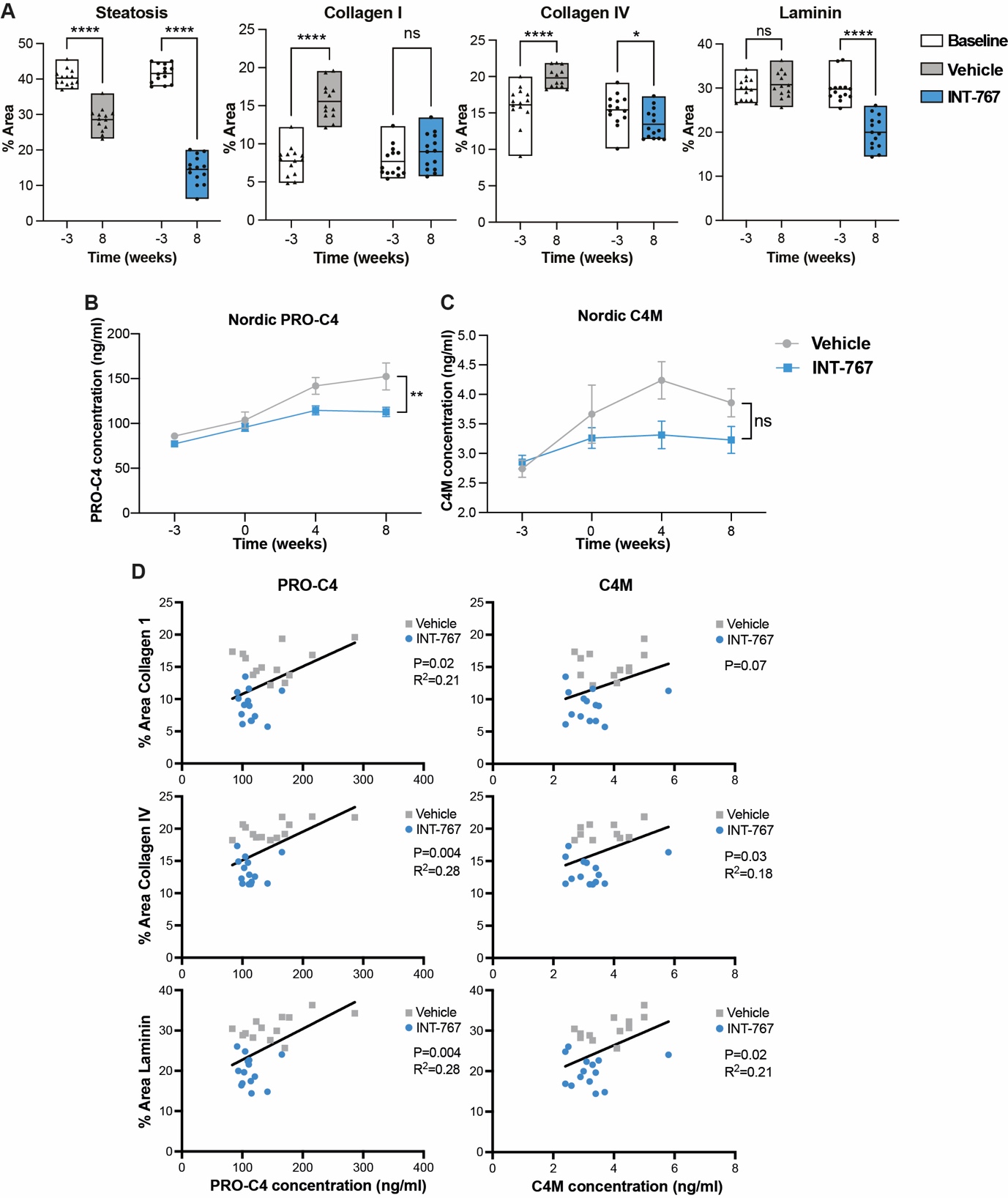
**

**Supplementary Fig. 2. Circulating biomarkers of basement membrane turnover are attenuated by INT-767 in AMLN *ob/ob*-MASH mice during MASH progression. (**A**)** Histological quantification of changes in % steatosis, type I collagen, type IV collagen and laminin staining in AMLN *ob/ob*-MASH mice between baseline biopsy (-3 weeks) and following 8 weeks treatment with vehicle (n=13) or INT-767 (n=14). Mixed-effects analysis with Šidák’s multiple comparison test. Levels of significance: ****p<0.0001, *p=0.013. (B) Serum PRO-C4 concentration in vehicle (n=13) and INT-767 (n=14) treated animals at stated time in injury model shown in Fig 1A. Levels of significance: ** p=0.0094 between groups (2-way ANOVA). (C) Serum C4M concentration in vehicle (n=13) and INT-767 (n=14) treated animals at stated time in injury model shown in Fig 1A. Levels of significance: n.s. p=0.117 between groups (2-way ANOVA mixed effects analysis). (D) Correlation of serum concentration of PRO-C4 or C4M with histological quantification of % type I collagen, type IV collagen or laminin staining in AMLN *ob/ob*-MASH mice following treatment with INT-767 (n=14 for PRO-C4 and C4M) or vehicle (n=13 for PRO-C4 and n=12 for C4M) in injury model shown in Fig 1A. Levels of significance stated (simple linear regression).

## SDC, Figure 3

**
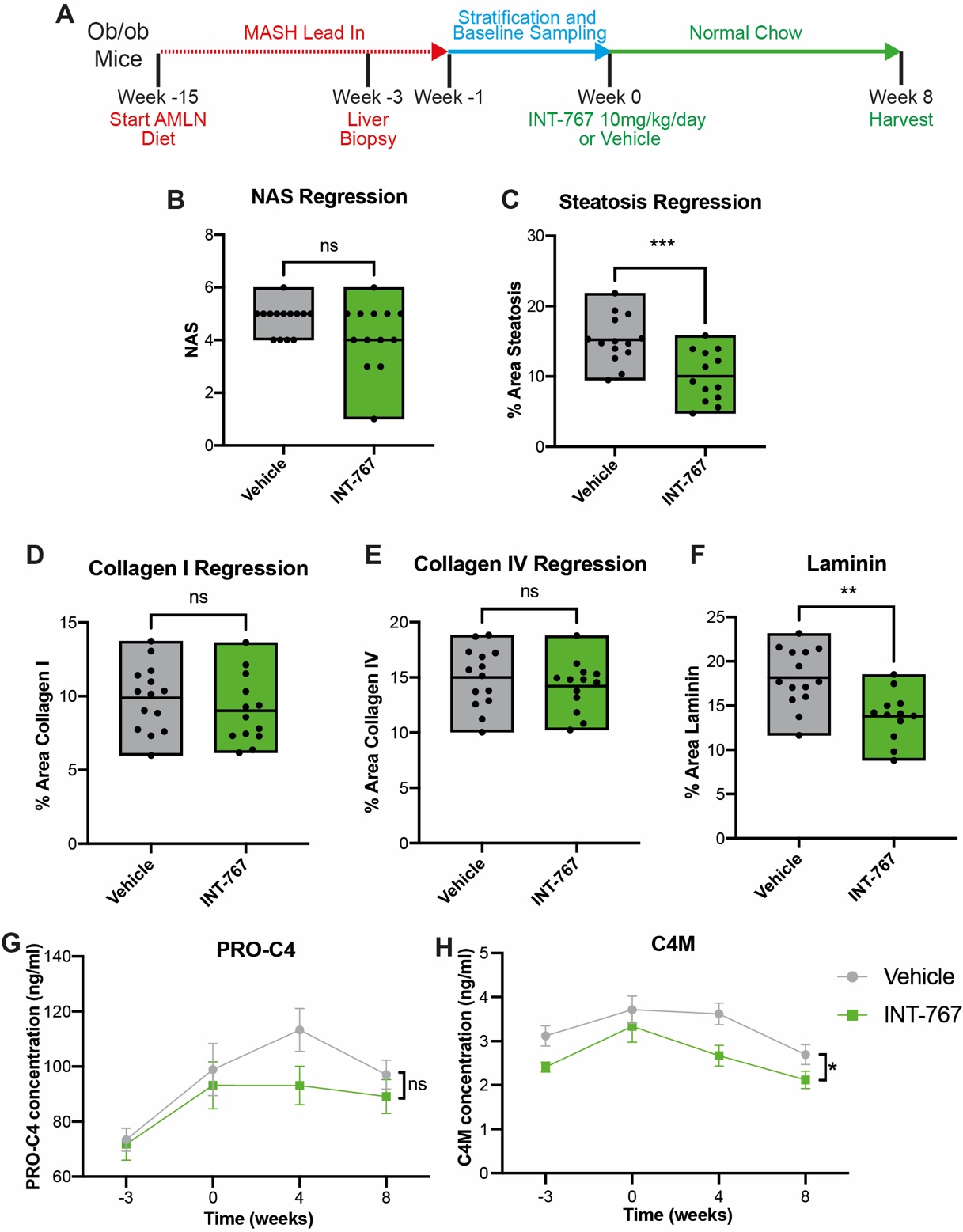
**

**Supplementary Fig. 3. INT-767 does not promote fibrosis regression during chow-reversal in AMLN *ob/ob*-MASH mice.** (A) Summary of AMLN *ob/ob*-MASH mouse chow-reversal model. Lep^ob/ob^ mice on AMLN diet for 12 weeks underwent liver biopsy. Mice with fibrosis stage ≥1 were converted to normal chow and were randomized to treatment with either INT-767 (10 mg/kg) or vehicle once daily for a further 8 weeks. (B) Histological quantification of NAS in AMLN *ob/ob*-MASH mice following treatment with INT-767 (n=13) or vehicle (n=14) during chow-reversal. Levels of significance: n.s. p=0.165 (Mann-Whitney test). (C) Histological quantification of % steatosis in AMLN *ob/ob*-MASH mice following treatment with INT-767 (n=13) or vehicle (n=14) during chow-reversal. Levels of significance: ***p=0.0008 (unpaired *t*-test). (D) Histological quantification of % type I collagen staining in AMLN *ob/ob*-MASH mice following treatment with INT-767 (n=13) or vehicle (n=14) during chow-reversal. Levels of significance: n.s. p=0.339 (unpaired *t*-test). (E) Histological quantification of % type IV collagen staining in AMLN *ob/ob*-MASH mice following treatment with INT-767 (n=13) or vehicle (n=14) during chow-reversal. Levels of significance: n.s. p=0.427 (unpaired *t*-test). (F) Histological quantification of % laminin staining in AMLN *ob/ob*-MASH mice following treatment with INT-767 (n=12) or vehicle (n=14) during chow-reversal. Levels of significance: ** p=0.0015 (unpaired *t*-test). (G) Serum PRO-C4 concentration in vehicle (n=14) and INT-767 (n=13) treated AMLN *ob/ob*-MASH animals at stated time during chow-reversal shown in Supplementary Fig 3A. Levels of significance: n.s. p=0.258 between groups (2-way ANOVA). (H) Serum C4M concentration in vehicle (n=14) and INT-767 (n=13) treated AMLN *ob/ob*-MASH animals at stated time during chow-reversal shown in Supplementary Fig 3A. Levels of significance: * p=0.047 between groups (2-way ANOVA mixed effects analysis).

## SDC, Figure 4


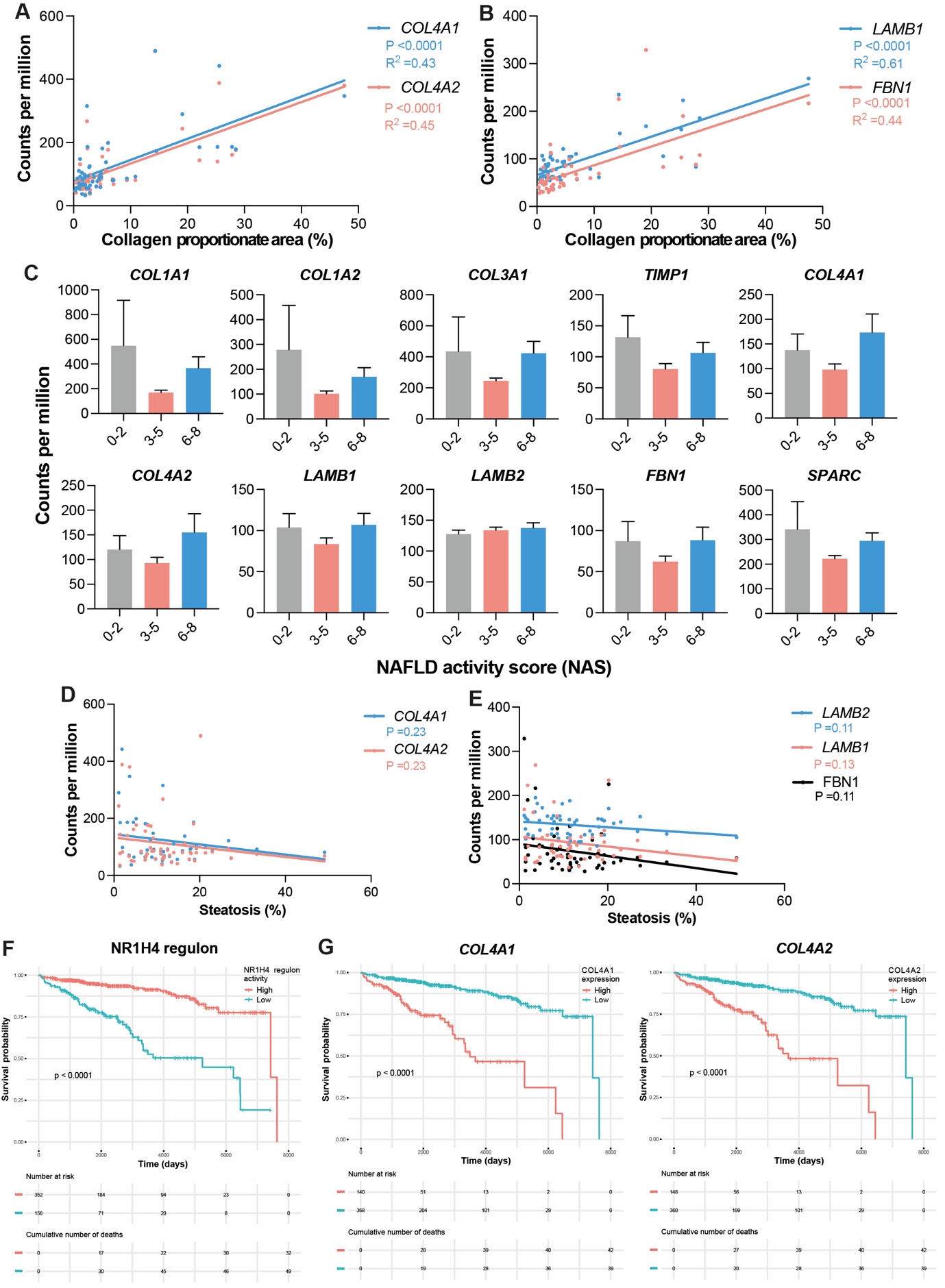


**Supplementary Fig. 4. Basement membrane expression does not correlate with NAFLD Activity Score (NAS) in human MASH.** (A-B) Correlation of expression of stated basement membrane genes from human MASH liver RNA-seq data with the degree of fibrosis quantified by collagen proportionate area (n=54). Levels of significance: p<0.0001 (simple linear regression). (C) RNA-seq analysis of human MASH liver tissue, grouped by histological NAS. Comparison of expression (in counts per million) of stated gene between NAS 0-2 (n=13), NAS 3-5 (n=30) and NAS 6-8 (n=11). Levels of significance: all comparisons p>0.05 (Welch’s ANOVA with Dunnett’s T3 multiple comparison test). (D-E) Correlation of expression of stated basement membrane genes from human MASH liver RNA-seq data with % steatosis (n=54). Levels of significance shown (simple linear regression). (F-G) Biopsy cases from the SteatoSITE dataset (n=508) with available RNAseq were used for time-to-event analysis. The NR1H4 (FXR) regulon activity profile (F) and normalized gene expression counts for COL4A1 and COL4A2 (G) were used to divide cases into low and high risk of death (Kaplan–Meier estimator curves with log-rank test p-value shown).

## SDC, Figure 5

**
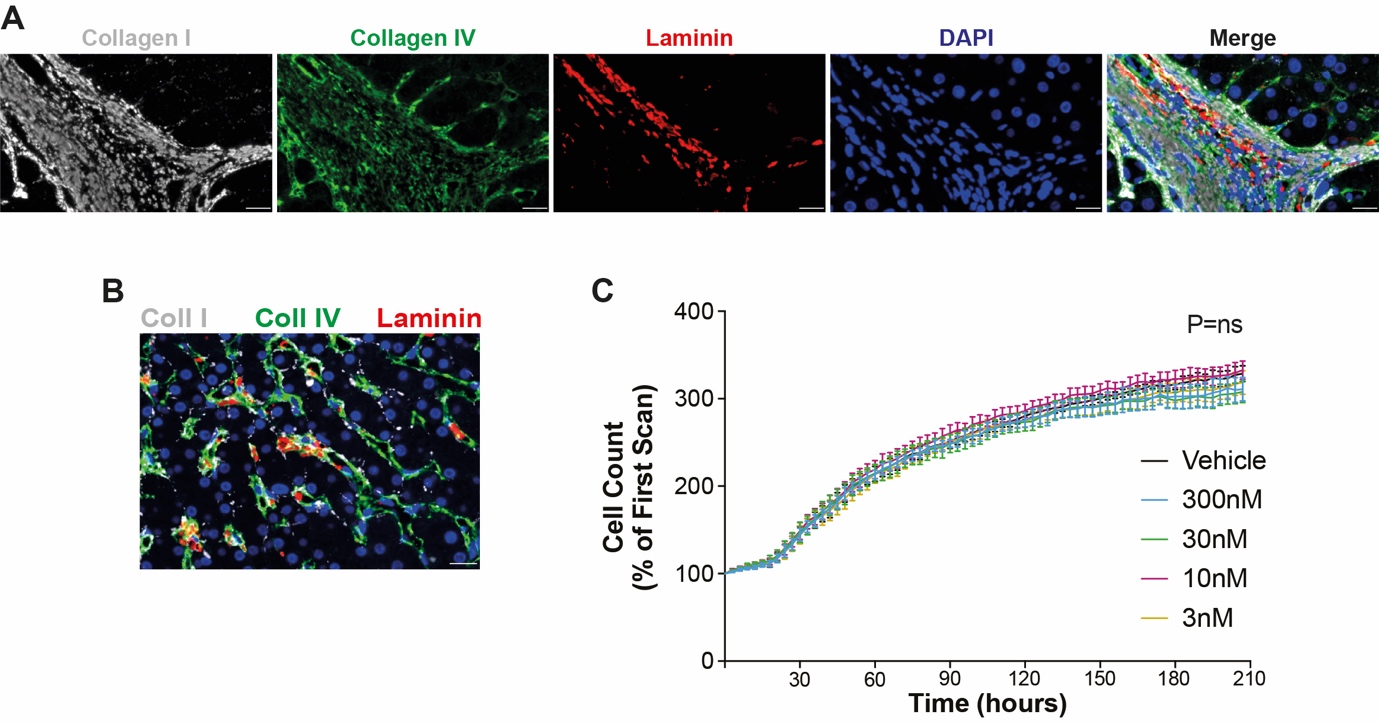
**

**Supplementary Fig. 5. Basement membrane components accumulate in the fibrotic niche of human MASH.**

(A) Representative immunofluorescence images of fibrotic niche of human MASH liver tissue for stated markers. Individual channel images and merged images shown. Scale bar = 20 μm. (B) Representative immunofluorescence image of parenchyma of human MASH liver tissue for stated markers. Merged channel image shown. Scale bar = 20 μm. (C) Proliferation of primary human HSCs *in vitro* in response to treatment with stated concentration of INT-767 (n=4) or vehicle (DMSO) control (n=4), assessed using Incucyte Zoom live cell imaging system. Cell counts were measured every 3 hours for 5 days and expressed as the percentage change in nuclei number from baseline. Levels of significance between INT-767 concentrations: n.s. p=0.758 (Two-way Repeated Measure ANOVA).

## SDC, Table 1

**Supplementary Table 1.** Immunohistochemistry antibodies used.

| Antibody | Company | Cat. Number | Concentration | Target Species |
| --- | --- | --- | --- | --- |
| Laminin | Dako | Z0097 | 1:50 | Mouse/Human |
| Col I | Southern Biotech | 1310-01 | 1:400 | Mouse/Human |
| Col IV | Southern Biotech | 1340-01 | 1:100 | Mouse/Human |
| FXR | Thermofisher Scientific | A9033A | 1:900 | Human |
| ⍺-SMA | Sigma | A5228 | 1:1000 | Human |

## SDC, Table 2

**Supplementary Table 2.** QuantiTect Primer Assays used for qRT-PCR.

## SDC, Table 3

**Supplementary Table 3.** List of differentially expressed genes from analysis shown in Fig 1B. Cluster 1 genes (downregulated in response to INT-767) and cluster 2 genes (upregulated in response to INT-767) are listed.

## SDC, References

1. Leeming DJ, Nielsen MJ, Dai Y, Veidal SS, Vassiliadis E, Zhang C, et al. Enzyme-linked immunosorbent serum assay specific for the 7S domain of Collagen Type IV (P4NP 7S): A marker related to the extracellular matrix remodeling during liver fibrogenesis. Hepatology Research. 2012;42:482–493.

2. Sand JM, Larsen L, Hogaboam C, Martinez F, Han M, Røssel Larsen M, et al. MMP Mediated Degradation of Type IV Collagen Alpha 1 and Alpha 3 Chains Reflects Basement Membrane Remodeling in Experimental and Clinical Fibrosis – Validation of Two Novel Biomarker Assays. PLoS One. 2013;8:e84934.

3. Roth JD, Feigh M, Veidal SS, Fensholdt LKD, Rigbolt KT, Hansen HH, et al. INT-767 improves histopathological features in a diet-induced ob/ob mouse model of biopsy-confirmed non-alcoholic steatohepatitis. World J Gastroenterol. 2018;24:195–210.

4. Dobin A, Davis CA, Schlesinger F, Drenkow J, Zaleski C, Jha S, et al. STAR: ultrafast universal RNA-seq aligner. Bioinformatics. 2013;29:15–21.

5. Robinson MD, McCarthy DJ, Smyth GK. edgeR: a Bioconductor package for differential expression analysis of digital gene  expression data. Bioinformatics. 2010;26:139–140.

6. Galili T. dendextend: an R package for visualizing, adjusting and comparing trees of hierarchical clustering. Bioinformatics. 2015;31:3718–3720.

7. Huang DW, Sherman BT, Lempicki RA. Bioinformatics enrichment tools: paths toward the comprehensive functional analysis  of large gene lists. Nucleic Acids Res. 2009;37:1–13.

8. Huang DW, Sherman BT, Lempicki RA. Systematic and integrative analysis of large gene lists using DAVID bioinformatics  resources. Nat Protoc. 2009;4:44–57.

9. Kendall TJ, Jimenez-Ramos M, Turner F, Ramachandran P, Minnier J, McColgan MD, et al. An integrated gene-to-outcome multimodal database for metabolic  dysfunction-associated steatotic liver disease. Nat Med. 2023;29:2939–2953.

10. Martin M. Cutadapt removes adapter sequences from high-throughput sequencing reads. EMBnet.journal; Vol 17, No 1: Next Generation Sequencing Data Analysis. 2011;

11. Liao Y, Smyth GK, Shi W. featureCounts: an efficient general purpose program for assigning sequence reads to  genomic features. Bioinformatics. 2014;30:923–930.

12. Robinson MD, Oshlack A. A scaling normalization method for differential expression analysis of RNA-seq data. Genome Biol. 2010;11:R25.

13. Wu D, Lim E, Vaillant F, Asselin-Labat M-L, Visvader JE, Smyth GK. ROAST: rotation gene set tests for complex microarray experiments. Bioinformatics. 2010;26:2176–2182.

14. Ritchie ME, Phipson B, Wu D, Hu Y, Law CW, Shi W, et al. limma powers differential expression analyses for RNA-sequencing and microarray  studies. Nucleic Acids Res. 2015;43:e47.

15. Ramachandran P, Dobie R, Wilson-Kanamori JR, Dora EF, Henderson BEP, Luu NT, et al. Resolving the fibrotic niche of human liver cirrhosis at single-cell level. Nature. 2019;575:512–518.

16. Korsunsky I, Millard N, Fan J, Slowikowski K, Zhang F, Wei K, et al. Fast, sensitive and accurate integration of single-cell data with Harmony. Nat Methods. 2019;16:1289–1296.

17. Xiong X, Kuang H, Ansari S, Liu T, Gong J, Wang S, et al. Landscape of Intercellular Crosstalk in Healthy and NASH Liver Revealed by Single-Cell Secretome Gene Analysis. Mol Cell. 2019;75:644-660.e5.

18. Dobie R, Wilson-Kanamori JR, Henderson BEP, Smith JR, Matchett KP, Portman JR, et al. Single-Cell Transcriptomics Uncovers Zonation of Function in the Mesenchyme during Liver Fibrosis. Cell Rep. 2019;29:1832-1847.e8.

19. Marwick JA, Elliott RJR, Longden J, Makda A, Hirani N, Dhaliwal K, et al. Application of a High-Content Screening Assay Utilizing Primary Human Lung  Fibroblasts to Identify Antifibrotic Drugs for Rapid Repurposing in COVID-19 Patients. SLAS Discov. 2021;26:1091–1106.
